# Versatile carbon nanoplatforms for cancer treatment and diagnosis: strategies, applications and future perspectives

**DOI:** 10.7150/thno.69628

**Published:** 2022-02-21

**Authors:** Lu Tang, Jing Li, Ting Pan, Yue Yin, Yijun Mei, Qiaqia Xiao, Ruotong Wang, Ziwei Yan, Wei Wang

**Affiliations:** 1 State Key Laboratory of Natural Medicines, Department of Pharmaceutics, School of Pharmacy, China Pharmaceutical University, 210009 Nanjing, P. R. China; 2 NMPA Key Laboratory for Research and Evaluation of Pharmaceutical Preparations and Excipients, China Pharmaceutical University, 210009 Nanjing, P. R. China

**Keywords:** Carbon materials, Cancer theranostics, Nanotechnology, Targeted drug delivery, Synergistic strategy

## Abstract

Despite the encouraging breakthroughs in medical development, cancer remains one of the principle causes of death and threatens human health around the world. Conventional treatment strategies often kill cancer cells at the expense of serious adverse effects or great pain, which yet is not able to achieve an effective cure. Therefore, it is urgent to seek for other novel anticancer approaches to improve the survival rate and life quality of cancer patients. During the past decades, nanotechnology has made tremendous progress in cancer therapy due to many advantages such as targeted drug delivery, decreased dosage-related adverse effects and prolonged drug circulation time. In the context of nanomedicine, carbon nanomaterials occupy very significant positions. Owing to their innate outstanding optical, thermal, electronic, and mechanic features, easy functionalization possibility and large surface for drug loading, carbon nanomaterials serve as not only drug carriers, but also multifunctional platforms to combine with diverse treatment and diagnosis modalities against cancer. Therefore, developing more carbon-based nanoplatforms plays a critical role in cancer theranostics and an update overview that summarizes the recent achievement of carbon nanomaterial-mediated anticancer theranostic approaches is of necessity. In this review, five typical and widely investigated carbon nanomaterials including graphene, graphdiyne, fullerene, carbon nanotubes and carbon quantum dots are introduced in detail from the aspect of treatment strategies based on both cancer cells and tumor microenvironment-involved therapeutic targets. Meanwhile, modern diagnostic methods and clinical translatability of carbon nanomaterials will be highlighted as well.

## 1. Introduction

According to the fresh statistics in 2021, cancer is still one of the most challenging diseases that causes a considerable mortality in contemporary society 1. This deadly ailment is usually characterized by uncontrolled production of malignant cancer cells, which grows in an unlimited way and disturbs the physiological functions of the host through their invasive spreading throughout the body 2. During the past decades, numerous therapeutic strategies have been evolved to address this dreadful disease. Conventional treatment modalities like chemotherapy, radiotherapy and surgical resection often fail to achieve an effective cure, which yet causes severe pain to cancer patients 3. Other emerging therapeutic strategies like immunotherapy, gene therapy and phototherapy largely patch up the limitations of conventional cancer treatment methods, however, these novel modalities also face other obstacles such as systemic side effects, allergic reactions, and high specificity to certain cancer types, which all impedes their extensive application in clinic 4. Moreover, when cancer develops into metastatic, its treatment regime becomes more complicated, expensive, and often ineffective. Therefore, novel technologies for early cancer detection, monitoring and focal lesion control are very essential to elevate the survival opportunity of cancer patients in advanced stage.

**Figure 1:**
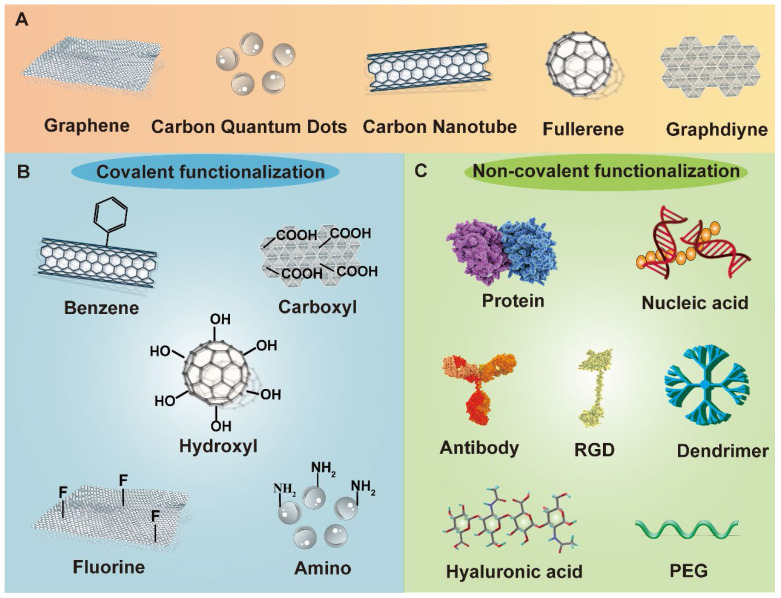
Schematic demonstration of different functionalized methods of carbon nanomaterials. Carbon nanomaterials can be modified both covalently and non-covalently through various chemical groups and biomolecules. The functionalization of carbon nanomaterials plays a vital role in improving anticancer therapeutic performance. Abbreviations: PEG, polyethylene glycol; RGD, Arginine-Glycine-Aspartate peptide.

Owing to the achievements in nanobiotechnology, application of nanomaterials in cancer theranostics is receiving immense attention 5-8. The combination of nanoplatforms with multiple anticancer agents open a new era for cancer treatment, which largely enhances the therapeutic efficacy while causing reduced adverse effects 9, 10. Among a variety of nanomaterials, carbon-based nanostructures stand out as one of the most attractive candidates in cancer theranostics due to their distinct physiochemical properties 11. A number of carbon-related nanoplatforms like graphene and its oxide, graphdiyne, fullerene and its derivatives, carbon nanotubes (CNTs), and carbon quantum dots (CQDs) have been broadly explored as drug carriers, photoactive and diagnostic agents in cancer theranostics 12, 13. Owing to their distinctive optical, electronic, thermal and mechanical features, multifunctional modification chemistry, large surface area, and more biocompatible profiles than metallic nanomaterials, carbon-based nanomaterials are gathering plenty of scientific attention in biomedical fields 14. Moreover, these carbonaceous nanostructures are inherently hydrophobic, therefore, therapeutic agents can be loaded on these carriers through hydrophobic interactions or π-π bonding, serving as effective drug delivery vehicles 15. Because of their easy functionalization possibilities, carbon-based nanomaterials can be modified with other biomolecules either covalently or non-covalently on their surface to increase their biocompatibility, biosafety and water solubility 16. In addition, many desired functionalized molecules or targeting ligands can also be incorporated into carbon nanomaterials to endow these platforms with active targetability or tumor homing capability 17 (**Figure 1**). More excitingly, the natural optical characteristics of carbon nanostructures make them indispensible and dependable materials in multi-mode anticancer theranostics, which is able to integrate targeted drug delivery, phototherapy, cancer imaging and other conventional treatment strategies in one platform, largely enriching the therapeutic field of multiple cancer treatment and diagnosis 18.

Effective management and precise elimination of malignant tumors require an accurate understanding of tumor features and the interplays between tumor cells and their surrounding microenvironment. Currently, many anticancer strategies are focusing on targeting tumor cells or the components in tumor microenvironment (TME) where tumor cells live. Targeting tumor parenchyma can directly kill tumor cells, while targeting TME elements is able to impact on their survival by disturbing their living environment, both playing key roles in developing anticancer strategies 19, 20. The versatility of carbon nanomaterials enables their multifunctionality in cancer treatment. These carbon-related carriers can deliver various anticancer agents to the intracellular sites of interest in cancer cells such as nucleus, mitochondria, cytoplasm and other organelles to realize direct tumoricidal effect 21, 22. Moreover, as TME is a very complicated system glutting with various cell types and thick extracellular matrix (ECM), which is featured by abnormal vasculature, acidity, high interstitial pressure, hypoxia, abundant glutathione (GSH) level, poor blood perfusion, and altered metabolism, thus, TME is considered as the soil for tumor progression and the bottleneck that limits the therapeutic efficacy of numerous cancer treatment approaches 23. Meanwhile, immunosuppressive properties of TME also facilitate tumor cells to escape immunotherapy, which largely hampers its therapeutic performance. Although the aforementioned obstacles due to TME produce a lot of impediments in cancer treatment, they can also be regarded as therapeutic targets for new strategy development. In light of this, many innovative anticancer methods including ECM modulation, anti-angiogenesis, cancer stem cells (CSCs) inhibitory, immunoregulatory, and TME-responsive controlled drug delivery that aim at remodeling TME have been widely studied.

**Figure 2:**
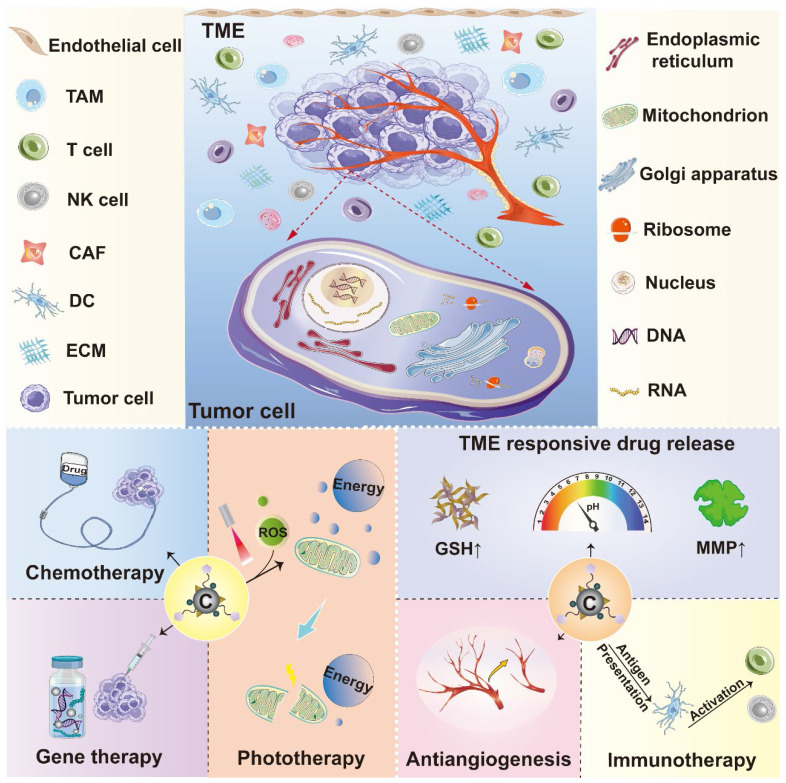
Schematic diagram illustrating the application of various carbon nanomaterials in cancer theranostics. Carbon nanomaterials can be adopted as effective tools to combine with many treatment modalities like chemotherapy, gene therapy, phototherapy and immunotherapy, which also serve as efficient drug carriers to target both cancer cells and the surrounding tumor microenvironment. Abbreviations: CAF, cancer-associated fibroblast; DC, dendritic cell; ECM, extracellular matrix; GSH, glutathione; MMP, matrix metalloproteinase; NK cell, natural killer cell; T cell, T lymphocyte; TAM, tumor-associated macrophage; TME, tumor microenvironment; ROS, reactive oxygen species.

Encouragingly, dedicated efforts have been devoted in the advancement of cancer nanomedicine. With the help of numerous researchers around the world, the field of carbon nanomaterials in cancer theranostics has been enriched with a lot of valuable pre-clinical data. In this review, we mainly summarized the recent achievements of carbon nanomaterials in cancer treatment and diagnosis. Different therapeutic strategies based on various carbon-related nanostructures aiming at a series of intracellular targeting spots or TME elements will be highlighted. In addition, diverse diagnostic approaches like various imaging technologies and cancer biosensors dependent on these carbonaceous nanomaterials will also be discussed (**Figure 2**). Altogether, compared to the existing papers related to this topic, the novelty of this review is that we comprehensively outlined the anticancer applications of various carbon-based nanoplatforms from the perspective of different therapeutic targets and focused on their theranostic applications against many cancer types based on fresh reported data.

## 2. Carbon nanomaterials for cancer treatment

Carbon-related nanomaterials are becoming important participants in cancer treatment and lots of anticancer strategies have been developed based on these nanomaterials. In this section, the contribution of various carbon nanomaterials including graphene, graphdiyne, fullerene, CNTs and CQDs to cancer treatment will be introduced. It is discussed how these carbon nanomaterials promote anticancer therapy through their natural physiochemical properties or combining with diverse therapeutic modalities. Summary of the application of various carbon nanomaterials in cancer treatment is listed in **Table 1**.

### 2.1 Graphene

**Figure 3:**
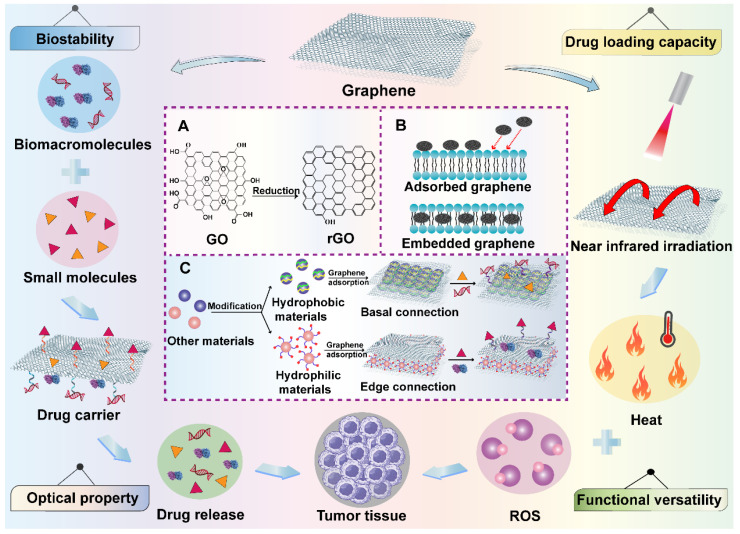
Schematic illustration of the functions of graphene-based nanomaterials in cancer treatment. Graphene-based nanomaterials can be combined with other materials to create new hybrid materials and can be utilized as excellent candidates in cancer treatment because of their good biostability, drug loading capacity, functional versatility and optical property. Abbreviations: GO, graphene oxide; rGO, reduced graphene oxide; ROS, reactive oxygen species.

Graphene-based nanomaterials have been broadly studied in biomedical territory for anticancer drug delivery since they were discovered in 2004 49. Graphene and its derivatives such as graphene oxide (GO), reduced graphene oxide (rGO) and graphene nanoribbons (GNR) have many unique and superior physicochemical properties, which are often designed as novel platforms to integrate with a lot of strategies like phototherapy, chemotherapy and bioimaging for cancer theranostics 50. Their two-dimensional (2D) structure provides a huge binding surface area in the hydrophobic basal plane, which enables the loading of various anticancer agents through hydrophobic interaction or conjugate reaction. Meanwhile, hydrophilic drugs can be non-covalently connected at the edges of graphene-based nanomaterials by electrostatic interaction and hydrogen bond 51. In addition, owing to the existence of easily modifiable and active oxygen-containing groups on their basal and edges, some functional molecules can be connected to them to achieve specific targeting for excellent antitumor effect 49. The sp^2^ hybridization of graphene-based nanomaterials endows them with a unique honeycomb lattice structure that results in their extraordinary electronic properties such as strong interactions between low-frequency photons and terahertz frequencies 52. Moreover, their exogenous light absorption spans from ultraviolet (UV) to infrared regions, especially in the near-infrared (NIR) regions, providing them with excellent optical properties under NIR irradiation. For instance, graphene and its derivatives can be stimulated by light energy to produce hyperthermia 53. Meanwhile, because of their capacity to carry a variety of photosensitizers and generate reactive oxygen species (ROS) under laser irradiation, typical photodynamic therapy (PDT) can be realized by graphene-based nanomaterials for efficient cancer elimination 54. Additionally, specific or controlled drug release are also feasible due to the trigger of light, which provides more chips for graphene-based nanomaterials to serve as intelligent drug delivery systems. Graphene and its derivatives also play significant roles in cancer immunotherapy due to their unique immune characteristics. It was reported that small particle size graphene is easier to activate immune cells, induces the release of cytokines and regulates the immune responses 55. Moreover, study has shown that the immunogenicity of graphene-based nanomaterials can be greatly improved through surface modification, which remarkably reduces their immunotoxicity and provides them with other excellent properties to enable their better application in cancer treatment 56. Except for the application of single nanomaterial components, graphene-based hybrid nanocomponents have been developed to overcome the limited function of graphene, which ameliorates their natural characteristics to achieve great microstructure and improved mechanical properties 57. For instance, various polymers are able to be coated on their surface to form a shell-core structure. In addition, graphene-based nanomaterials can also be directly embedded into the body of other nanomaterials like silicone rubber, cell membrane through π-π or hydrogen bond to form a brand-new polymer, which not only retains the excellent features of the applied nanomaterials, but also makes full use of the unique advantages of graphene-based nanomaterials 58. Therefore, due to the outstanding properties mentioned above, graphene-based nanomaterials are very promising candidates in cancer therapy and diagnosis (**Figure 3**).

#### 2.1.1 Graphene for intracellular target-based anticancer strategies

Guo *et al.* fabricated a novel nanoplatform using PEGylated and oxidized sodium alginate (OSA)-modified GO nanosheets to load anticancer drug paclitaxel (PTX) for PTX-resistant gastric cancer treatment, which could achieve synergistic chemotherapy/PTT/PDT effect 24. As P-glycoprotein (P-gp) is a key factor that is responsible for developing drug resistance, which can pump PTX out of gastric cancer cells, therefore, suppression of the out-pumping function of P-gp might be a possible approach to decrease drug resistance 59. Different from downregulating the expression of P-gp as reported, this proposed GO-based nanoplatform could suppress the energy supply of P-gp by inducing the depolarization of mitochondrial transmembrane potential (MTP) to cause mitochondrial damage upon NIR irradiation. The generated ROS through PDT could attack the respiratory chain complex enzymes of mitochondria and reduce the ATP supply of P-gp, thus effectively inhibiting the efflux pump function of P-gp and reversing the drug resistance of PTX. The cellular uptake results showed that this GO-based nanocomposite could be detected in the cytoplasm of HGC-27/PTX cancer cells without similar observations in control groups, which implied the excellent intracellular targeting ability of this nanocomposite. More excitingly, it was confirmed *in vitro* and *in vivo* that this newly prepared nanocomposite could lead to enhanced cytotoxicity upon NIR irradiation than free PTX in the gastric cancer model, demonstrating a promising therapeutic strategy to reverse gastric cancer cell resistance by combining chemotherapy and phototherapy.

Antitumor therapeutic strategies targeting microRNAs (miRNAs) have become a hot spot for decades. Among them, miR-214 can promote oral squamous cell carcinoma (OSCC) cell proliferation by regulating multiple signaling pathways 60. Electrostatic repulsion exists between GO and nucleic acid because they are both negatively charged, while polyetherimide (PEI) is a positively charged gene vector with a proton sponge effect, which can reduce the electrostatic repulsion between miRNAs and GO, thus improving the overall stability 61. Ou* et al.* firstly reported a gene therapy strategy for OSCC treatment by using positively charged PEI-functionalized GO to transport the inhibitor of miR-214 for suppressing OSCC cell proliferation 25. *In vitro* and *in vivo* results showed that this nanocarrier could remarkably inhibit Cal27 and SCC9 tumor cell growth and migration by targeting phosphatase and tensin homolog (PTEN) and p53. Besides,* in vitro* experiments proved that GO-PEI was able to elevate the transfection efficiency of miR-214 inhibitor in Ca127 and SCC9 cells, while miR-214 inhibitor alone was not capable of penetrating the membrane of tumor cells, and the transfection rate was 10 times higher than that of naked inhibitor, verifying GO as an ideal carrier for intracellular delivery of miRNA. Jaleel *et al.* designed a targeted antitumor gene therapy strategy using GO-based non-viral vectors 26. Briefly, this gene vector was constructed by attaching plasmid deoxyribonucleic acid (pDNA)-TNF-α to chitosan-carboxylated GO through electrostatic interaction, which was followed by polyethylene glycol (PEG) coating to prolong their circulation time, and then the folic acid (FA) derived carbon dots (C-dots) was modified on the delivery system to achieve active targeting due to the overexpressed folate receptors on tumor cells. This newly prepared gene delivery vector could actively target tumor cells and deliver pDNA into their nucleus to affect the expression of TNF-α. *In vitro* protein expression study successfully proved that this GO-based delivery system could improve gene transfection efficiency compared to pDNA-TNF-α alone. In addition, after 14 days of co-incubation with the chorioallantoic membrane (CAM) and Hela cells, the anti-angiogenesis effect was observed in GO-based formulation while no similar phenomenon could be found in the control group.

#### 2.1.2 Graphene for TME-based anticancer strategies

**Figure 4:**
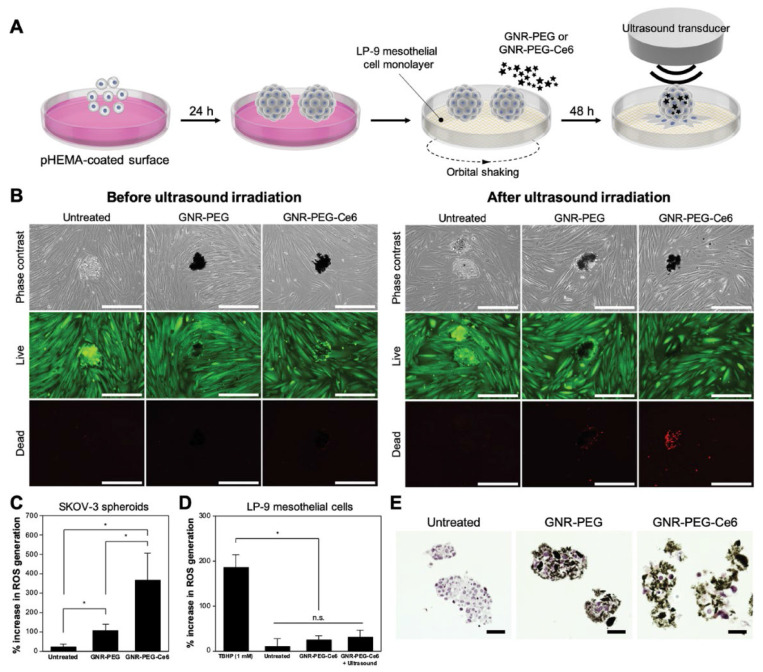
GNR-PEG-Ce6 can kill adhered ovarian cancer spheroids via sonodynamic therapy. **(A)** Schematic of the process for ultrasound irradiation of adhered spheroids. **(B)** Representative images of live (green) and dead (red) cells in untreated, GNR-PEG-treated, and GNR-PEG-Ce6-treated SKOV-3 spheroids adhered to the LP-9 mesothelial cell layer before and after ultrasound irradiation. Scale bars indicate 400 μm. ROS generation in **(C)** SKOV-3 spheroids and **(D)** LP-9 mesothelial cells after ultrasound irradiation (∗*p <* 0.05). Tert-butyl hydrogen peroxide (TBHP) was used as a positive control in this assay. **(E)** Hematoxylin and eosin-stained (histological) cross-sections of untreated, GNR-PEG-treated, and GNR-PEG-Ce6-treated SKOV-3 spheroids. Scale bars indicate 50 μm. Reproduced with permission from 29, copyright 2021, Wiley-VCH.

Immune cells are very important constituents of TME. The dynamic interactions between immune cells and other cell types within TME play vital roles in tumorigenesis, thus, anticancer strategies focusing on immunotherapy are of great significance. Messenger RNA (mRNA) vaccine generally refer to the mRNA which can activate dormant T cells for immune detection and automatically kill abnormal tumor cells. These mRNAs can get ingested by antigen-presenting cells (APCs) and express tumor-associated antigens (TAAs), thus activating CD4^+^ and CD8^+^ cells in TME to perform cellular or humoral immune responses to produce killing effects on tumors 62. However, these mRNA vaccines can only be expressed in specific immune cells or lymphatic vessels, and are easy to be destroyed due to their poor stability, therefore, they cannot transmit TAAs continuously. To address the aforementioned obstacles, Yin *et al.* synthesized a nanovaccine with sustained-release behavior based on hydrogel, which was composed of PEI modified GO to encapsulate ovalbumin (OVA) encoding mRNA (mOVA) and hydrophobic immune adjuvants Resiquimod (R848) for melanoma treatment 27. This nanocarrier could deliver mOVA and R848 to lymph nodes to elevate the amount of CD8^+^ T cells as well as produce antigen-specific antibodies and inflammatory factors such as TNF-α. The Western blot analysis demonstrated that OVA proteins were highly expressed in RAW264.7 and DC2.4 cells treated with GO-based hydrogel, indicating that the mOVA vaccine could be successfully expressed. The B16-OVA melanoma model was established to testify the antitumor effect of the hydrogel *in vivo.* As the obtained results showed, the tumor size and weight were significantly decreased compared to other groups, which demonstrated that the mRNA vaccine could activate immune cells to produce the antitumor effect. Moreover, OVA-specific antibodies were found in the serum after treatment with GO-based hydrogel and the formation of lung metastasis were significantly prevented as observed, both results further proving the bright application prospect of mRNA vaccine in antitumor therapy. Strong aggressiveness and extremely rapid tumor cell proliferation are key challenges during the ovarian cancer treatment in advanced stage. Lee* et al.* brought a fresh tumor-targeted therapeutic strategy by decorating a sonosensitizer chlorin e6 (Ce6) and 4-arm PEG with good compatibility on the GNR to construct GNR-PEG-Ce6 nanocomplexes for metastatic ovarian cancer treatment 29. The constructed nanocomplexes could absorb onto the SKOV-3 tumor spheroids and reduce their adhesion to ECM proteins or LP-9 mesothelial cells, which delayed the disaggregation and spreading of tumor spheroid, as well as slowed down the mesothelial clearance which is a crucial metastatic process after adhesion. Moreover, due to the localized delivery of sonodynamic agent Ce6, the adhered ovarian cancer spheroids could be effectively eliminated by these as-prepared nanocomplexes upon mild ultrasound irradiation. More excitingly, when validating the samples derived from patient ascites, the efficacy of these nanocomplexes was also satisfactory, illustrating the translational possibility of graphene-based nanomaterials for attenuating ovarian cancer metastasis in clinic** (Figure 4)**.

**Figure 5:**
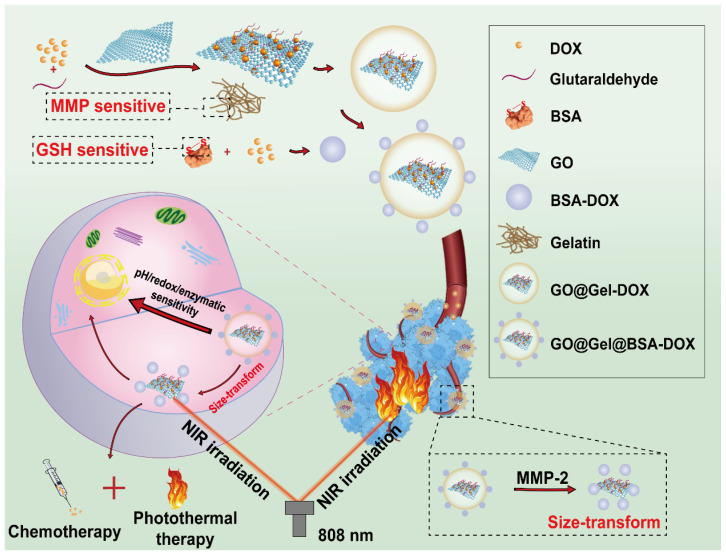
A diagram that illustrates the design and mechanism of GO@Gel@BSA-DOX nanohybrids for TME-responsive drug release and anticancer therapy. The constructed nanohybrids were pH/redox/enzyme sensitive and size-transformable, which showed outstanding therapeutic efficacy in combined chemo/photothermal therapy. Abbreviations: MMP, matrix metalloproteinase; GSH, glutathione; DOX, doxorubicin; BSA, bovine serum albumin; GO, graphene oxide; NIR, near-infrared Adapted with permission from 28, copyright 2021, Royal society of chemistry.

Because of the abnormal metabolic properties of tumor cells, TME is often featured as weakly acidic, high GSH amount and overexpressed matrix metalloproteinase-2 (MMP-2) level, therefore, many efforts have been made to design intelligent and responsive delivery system based on the internal properties of TME for efficient drug delivery 63. With this aim in mind, Wu *et al.* fabricated a pH/redox/enzymatic sensitive nanohybrid drug delivery system by assembling GSH sensitive bovine serum albumin (BSA) encapsulated DOX and MMP-2 sensitive gelatin onto GO nanosheets to achieve controlled drug release (**Figure 5**) 28. Under normal physiological conditions, this constructed nanosystem could maintain its stability, however, when it entered into TME, 5 nm nano-units encapsulating DOX could be released from this nanosystem due to the trigger of proteases which are highly expressed in TME. Moreover, after reaching the acidic, reductive and enzymatic tumor tissue, a synergistic chemo/photothermal therapeutic effect could be realized because of the switchable release of DOX upon NIR laser irradiation. As the *in vitro* photothermal experiments showed, this novel nanosystem could reach to 45.6 °C after irradiating for 5 min, which was high enough to ablate MCF-7 cells.

### 2.2 Graphdiyne

**Figure 6:**
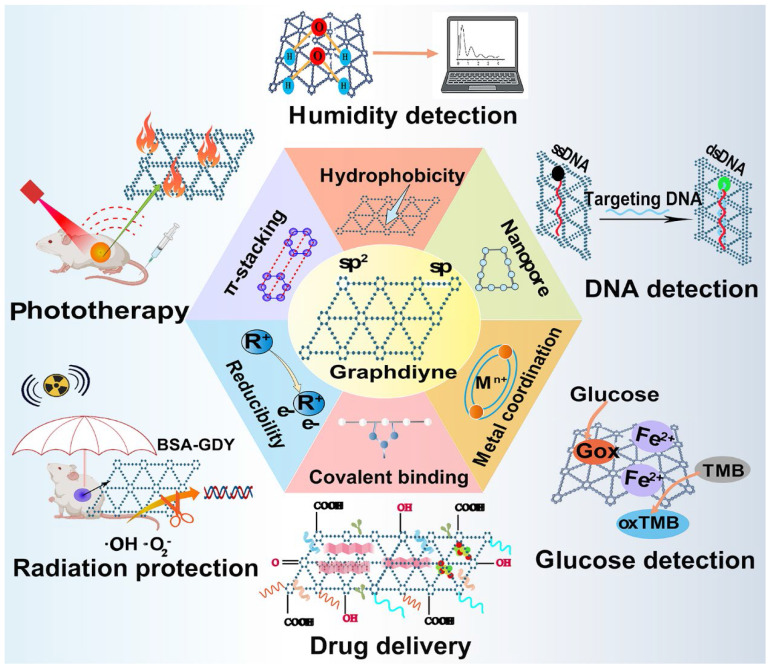
Current applications of graphdiyne-based nanomaterials in biomedical fields. Graphdiyne serves as an ideal candidate in many theranostic arena such as phototherapy against cancer, drug carrier, mediators in humidity, DNA and glucose detection and radiation protection, exhibiting great research potential in biomedical area. Abbreviations: GDY, graphdiyne; BSA, bovine serum albumin; ssDNA, single-stranded DNA; dsDNA, double-stranded DNA; Gox, glucose oxidase; TMB, 3,3',5,5'-tetramethylbenzidine; oxTMB, oxidized 3,3',5,5'-tetramethylbenzidine.

Graphdiyne (GDY), a 2D periodic material experimentally synthesized firstly in 2010, has become a kind of emerging carbon-related nanomaterial since its discovery 64. Unlike other carbon nanomaterials, GDY is characterized by assembled sp and sp^2^-hybridized planar structure with uniformly distributed nanopores, broad absorption range, and great photoelectronic features. The fundamental structural unit to constitute GDY is a big triangular ring that contains eighteen carbon atoms composed of benzene rings with sp^2^-hybridization and acetenyl groups with sp-hybridization 65. The acetylenic bonds are regarded as potential active sites, thus, GDY can be functionalized via the covalent or non-covalent interaction between GDY and unsaturated ligands. In contrast to other sp^2^-hybridized carbon nanomaterials, the conjugated structure of GDY can still retain after functionalization, meanwhile, the sp-hybridized carbon atom also endows GDY with reducibility 66. Moreover, active groups like carboxyl, hydroxyl and ketone groups is able to be attached on the surface of GDY to introduce more properties and broaden its application 33. Notably, by means of altering the layer numbers and stacking manners, GDY can be given with the ability to tune band gap, thereby offering more possibilities for generating novel GDY-based nanosystems with tunable electronic and optical properties 67. Currently, GDY and its derivatives are utilized as attractive metal-free semiconductor materials, energy storage material, catalyst, and free radical scavenger 68. In addition, the distinctive macroporous structure of GDY with high degree of π-π conjugations provides it with strong adsorption capacity for many therapeutic molecules 65. Therefore, the presence of sp^2^-hybridized structure and specific surface area make GDY an ideal delivery vehicle to transport a number of anticancer agents including genes, small molecule drugs, functional polymers, and biomacromolecules through π-π stacking, hydrophobic and electrostatic interaction between drugs and vehicles 69. Moreover, GDY can also serve as a biosensor for DNA, glucose, and humidity detection due to its high adsorption energy and strong electron capturing properties 70. Besides, given the broad absorption throughout the visible region, GDY can act as a photothermal agent to induce hyperthermia and serve as photoacoustic imaging (PAI) contrast agent in cancer imaging, whose photothermal conversion efficiency is higher than other classic PTT agents 71. So far, GDY-based nanomaterials have been widely explored in cancer theranostic field including targeted drug delivery, radiation protection, biosensing and bioimaging, cancer therapy, which hold advantages over traditional carbon nanomaterials because of their multifunctional performance (**Figure 6**) 65.

#### 2.2.1 Graphdiyne for intracellular target-based anticancer strategies

**Figure 7:**
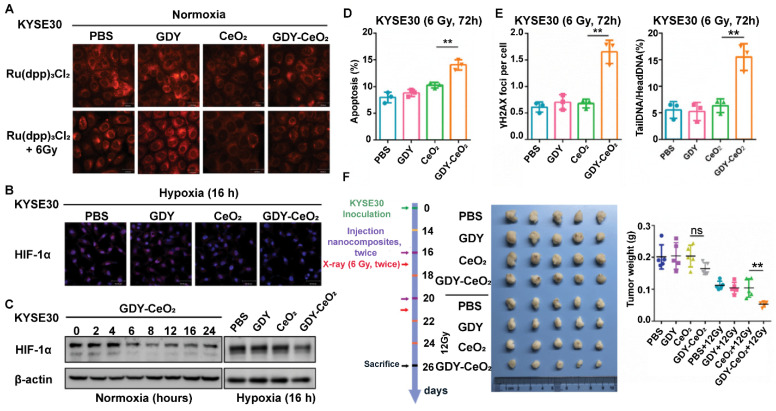
GDY-CeO_2_ nanoparticles can attenuate hypoxia and enhance radiosensitivity. **(A)** KYSE30 cells were treated with GDY, CeO_2_, GDY-CeO_2_, or PBS for 4 h, and then treated with or without 6 Gy of X-rays. Intracellular O_2_ detection by [Ru(dpp)_3_]Cl_2_ (red) using confocal microscope. **(B)** KYSE30 cells were treated with GDY, CeO_2_, GDY-CeO_2_, or PBS under hypoxic conditions for 16 h. Red and blue fluorescence show HIF-1*α* expression and Hoechst-33342-stained nuclei in fixed cells with 4% paraformaldehyde, respectively. **(C)** KYSE30 cells were treated with GDY, CeO_2_, GDY-CeO_2_, or PBS under normoxic or hypoxic conditions for 16 h, and then HIF-1*α* protein expression was detected by western blot. **(D-E)** KYSE30 cells were treated with GDY, CeO_2_, GDY-CeO_2_, and PBS, respectively. **(D-E)** After 72 h, apoptosis was detected by flow cytometry **(D)**, *γ*H2AX foci and single cell gel electrophoresis for DNA damage **(E)** was performed. The data are presented as the mean ± standard deviation (SD). **(F)** Schematic illustration of therapeutic experiments in subcutaneous tumor models. Tumor weights were analyzed after the indicated treatments. *n* = 5 mice per group. The data are presented as the mean ± SD. Statistical analysis was performed by a two-tailed, unpaired Student's *t*-test, **p < 0.01. Reproduced with permission from 30, copyright 2021, Wiley-VCH.

**Figure 8:**
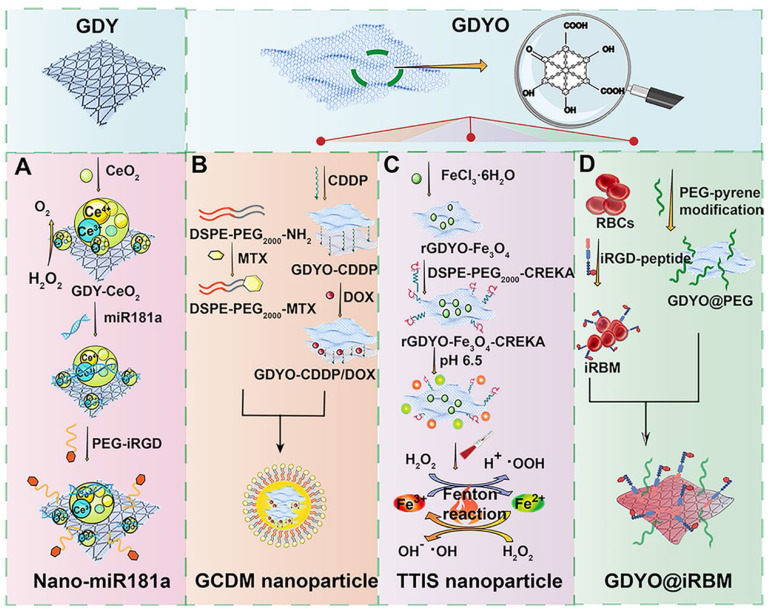
Schematic demonstration of the preparation of various graphdiyne-based nanosystems in cancer treatment. Construction of **(A)** Nano-miR181a complexes. Adapted with permission from 30, copyright 2021, Wiley-VCH, **(B)** GCDM nanoparticle. Adapted with permission from 31, copyright 2021, Wiley-VCH, **(C)** TTIS nanoparticle. Adapted with permission from 32, copyright 2020, Wiley-VCH, and **(D)** GDYO@iRBM nanosystem. Adapted with permission from 33, copyright 2019, American Chemical Society. Abbreviations: GDY, graphdiyne; GDYO, graphdiyne oxide; PEG, polyethylene glycol; iRGD, internalizing RGD peptide (CRGDKGPDC); CDDP, cisplatin; MTX, methotrexate; RBCs, red blood cells.

miRNAs regulate DNA damage response that is beneficial to genome integrity and stability, yet there are still existing obstacles that impede the wide application of miRNA-based therapy, such as internal degradation and rapid blood clearance. To overcome these limitations, lots of multifunctional nanosized carriers have been developed to prevent miRNAs from degradation, enhance tumor targeting and endow miRNAs with lysosomal escape ability 72. Radioresistance is another tricky challenge that limits the antitumor therapeutic efficacy in clinic, which is resulted from tumor hypoxia, improved DNA repair function, and the existence of CSCs 73. To address the two issues above, Zhou *et al.* developed a GDY-based nanoplatform that firmly anchored and dispersed CeO_2_ nanoparticles (NPs) to form GDY-CeO_2_ nanocomposites, which displayed excellent catalase activity due to CeO_2_ NPs, remarkably relieving tumor hypoxia through the decomposition of H_2_O_2_ into O_2_ (**Figure 8A**) 30. Moreover, miR181a, a miRNA-based formulation, which was capable of targeting RAD17 and regulating the Chk2 pathway to induce DNA damage and apoptosis, was loaded on GDY-CeO_2_ nanocomposites to elevate the sensitivity of radiotherapy against esophageal squamous cell carcinoma (ESCC). Besides, iRGD-grafted PEG was employed to encapsulate the constructed GDY-CeO_2_-miR181a to achieve enhanced tumor targeting and penetration, as well as protecting miRNA from degradation. *In vitro* and* in vivo* results illustrated that the designed nanocomposites were able to facilitate DNA damage and significantly downregulate the HIF-1α expression level by relieving the hypoxic tumor environment. Crucially, delivery of miR181a through GDY-CeO_2_ nanoplatform showed a remarkable efficacy in sensitizing tumor upon radiotherapy based on subcutaneous tumor and ESCC PDX models, which provided a prospective therapeutic strategy for personalized ESCC treatment (**Figure 7**).

Targeted synergistic therapy has become the research focus of cancer therapy at the present. Xing *et al.* established a multifunctional 3D carrier based on modified graphdiyne oxide (GDYO), which was hybridized with cisplatin (CDDP) to load DOX via π-π stacking and integrated cancer diagnosis and photo-chemotherapy because of the fluorescent characteristic of DOX (**Figure 8B**) 31. Moreover, in order to target folate receptors overexpressed on many tumor cell surfaces, this nanocarrier was further modified with methotrexate (MTX), which not only shares a similar structure like FA to realize active targeting, but also serves as a classic chemotherapeutic agent for tumor inhibition 74. Ultimately, this designed nanodrug carrier (termed as GCDM) achieved excellent synergistic photo-chemotherapy effect due to the outstanding optical features of GDYO and combinational regime of multiple chemotherapeutics. Both near-infrared fluorescent (NIRF) and fluorescent images confirmed the effective enhancement of drug accumulation and active targetability towards tumor sites when using GCDM. Experimental results obtained from Hela-tumor model demonstrated that the constructed GCDM could result in significant antitumor effectiveness without causing obvious toxicity, indicating the great biocompatibility and biosafety of this nanocarrier.

#### 2.2.2 Graphdiyne for TME-based anticancer strategies

ROS-mediated oncotherapy has gathered much attention because of its high selectivity and low adverse effects. Fenton reaction-based strategy is an emerging anticancer treatment approach, which can provide a catalyst to convert endogenous H_2_O_2_ into another member of ROS, namely hydroxyl radicals. However, the efficiency of Fenton reaction is always restricted by insufficient delivery of catalytic agents, unsuitable pH level and excess GSH in TME 75. To overcome these obstacles, Min *et al.* designed a tumor targeted iron sponge (TTIS) nanoplatform by depositing Fe_3_O_4_ NPs onto the surface of GDYO, which was further decorated with tumor targeting polymer to improve its biocompatibility and targetability (**Figure 8C**) 32. Besides, this fabricated nanoplatform could produce excellent photothermal performance to generate heat in TME, which helped to promote the efficacy of Fenton reaction through the accelerated release of Fe^3+^ and Fe^2+^ from TTIS, achieving the goal of synergistic PTT and Fenton reaction-mediated anticancer strategy. Both *in vivo* and *in vitro* experiments based on 4T1 tumor model confirmed the excellent antitumor effect of this nanoplatform with negligible toxicity, implying the great translational potential of this biocompatible nanoplatform in clinic. The poor vascular perfusion in hypoxic TME can contribute to tumor development, metastasis, and drug resistance. Rationally, tumor reoxygenation and blood perfusion enhancement are able to improve the therapeutic effect in cancer treatment. In light of this, Jiang *et al* reported a biomimetic ultrathin GDYO nanosheet which was cloaked with iRGD peptide-engineered erythrocyte membrane (termed as GDYO@i-RBM) (**Figure 8D**) 33. This biomimetic nanosystem could produce singlet oxygen by catalyzing water oxidation under NIR irradiation, which not only possessed excellent PTT effect, but also achieved synergistic PDT effect through the alleviation of tumor hypoxia and improvement of blood perfusion. Both* in vivo* and *in vitro* studies based on EMT-6 tumor models verified the markedly enhanced antitumor efficacy after the combination of GDYO@i-RBM plus laser irradiation, providing new insights in relieving tumor hypoxia through GDYO-based novel nanoplatform. Apart from the examples mentioned above, other TME-based strategies like designing pH/photo-dual responsive GDY nanosheet that could achieve controlled drug release in acid TME upon laser irradiation, or combining magnetic targeting with GDY-based therapeutic strategies also display promising research potential in cancer treatment 65.

### 2.3 Fullerene

**Figure 9:**
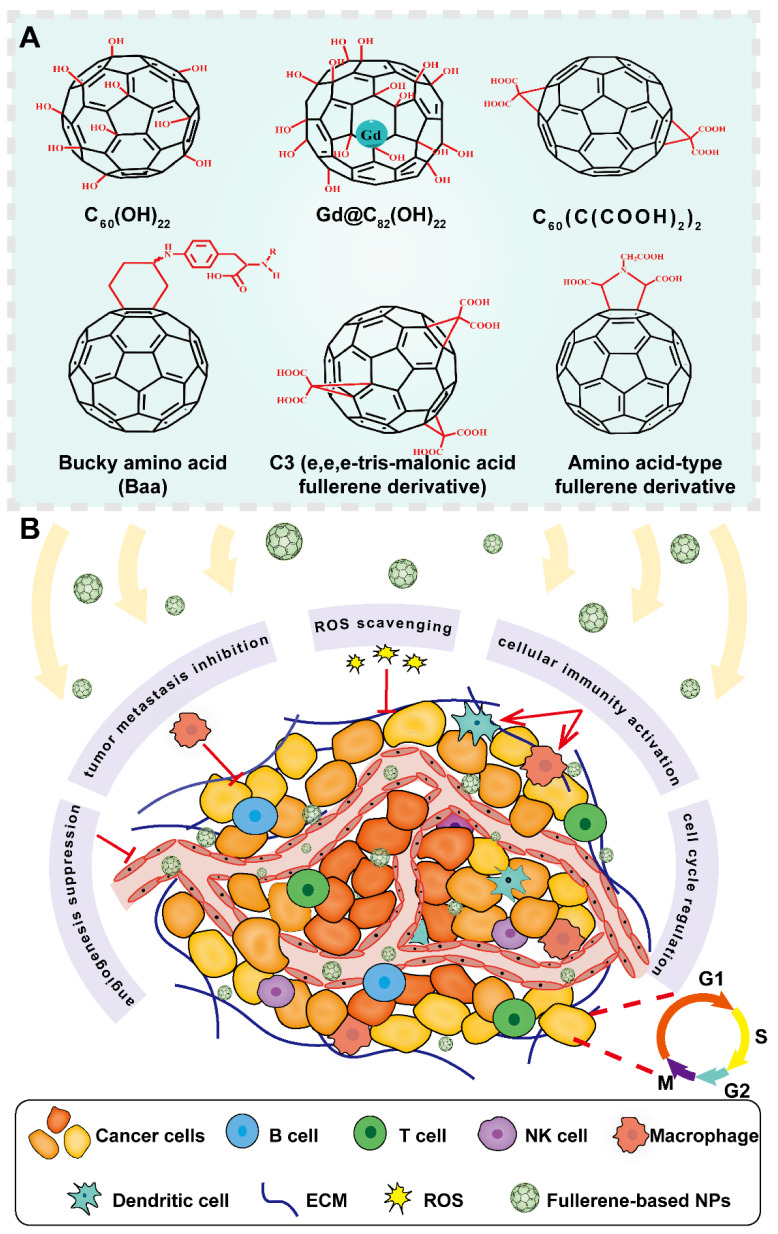
Representative structures of fullerene derivatives and their functions in cancer treatment. Fullerene-based nanosystems act as excellent candidates in cancer theranostics, showing diverse functions against cancer development including angiogenesis suppression, metastasis inhibition, ROS scavenging, immune activation and cell cycle regulation. Abbreviations: Gd, gadolinium; B cell, B lymphocyte; T cell, T lymphocyte; NK cell, natural killer cell; ECM, extracellular matrix; ROS, reactive oxygen species; NPs, nanoparticles.

Fullerene was discovered as carbon allotropes by Kroto *et al.* in 1985, who was awarded with Nobel prize of chemistry in 1996 for their amazing discovery 76. Since then, fullerene has been widely explored in cancer theranostics because of its special structures and distinct physiochemical characteristics. Fullerene is viewed as a form of graphene sheet that rolled into a hollow sphere, ellipsoid, or tube shape. It entirely consists of 60 carbon atoms linked by 30 carbon double bonds with abundant π-π conjugation in cage-type structure 11. Interestingly, the unique geometry and molecular topology of fullerene endow it with prominent properties such as large surface area, small particle size, and high reactivity. Furthermore, fullerene also exhibits electronic, optical, thermal, mechanical properties, and other versatile physicochemical features 77. The large specific area and unique 3D scaffold structure of fullerene enable the loading of multiple drugs in the fullerene cage through covalent bonding 11. Given the excellent characteristics of penetration into solid tumors and high chemical reactivity, fullerene can also be designed as stimuli-responsive systems based on the special conditions of the tumor sites to achieve targeted delivery of therapeutic molecules 78. In addition, due to its inherent optical and thermodynamic properties, fullerene has been proved to act as a good photosensitizing agent, making it a promising nanomaterial in cancer imaging, hyperthermia, PDT, and photoacoustic/thermoacoustic assisted theranostics 79. Notably, owing to its anticancer activity and sensitization effect on cancer cells, fullerene can serve as a strong antineoplastic agent 80. Besides, fullerene can be employed as an antioxidant due to its free radical scavenging capacity 80. Interestingly, metal atoms can also be incorporated into fullerene to generate metallofullerene, which inherits the combined properties from carbon cages and internal metal, showing promising prospects for application as contrasts in magnetic resonance imaging (MRI) and X-ray, radiotracers and anticancer agents 81. Taken together, fullerene and its derivatives can be tailored to meet specific demands of multifarious modalities in cancer treatment and diagnosis (Figure 9). Nevertheless, there are still several issues that limit the application of fullerene, such as poor water solubility and intrinsic toxicity. To cope with these limitations, fullerene can be covalently modified with water-soluble functional biopolymers to enhance its water solubility and tumor homing capability. Regarding its intrinsic toxicity, fullerene can be modified with biocompatible molecules to enhance their biosafety. For instance, many polar functional groups such as hydroxyl, carboxyl and amino groups can be attached on its surface to enhance its biocompatibility 80. Therefore, many fullerene derivatives like hydroxyfullerenes, carboxyfullerenes and glycofullerenes have emerged, which displays outstanding performance against cancer in multiple angles including angiogenesis inhibition, immune system activation, antioxidation and reversal of multidrug resistance 80.

#### 2.3.1 Fullerene for intracellular target-based anticancer strategies

It is well known that intracellular signaling pathways and metabolic processes are vital players in oncogenesis, tumor progression, and neoplastic dissemination. Therefore, based on the unique advantages of fullerene and its derivatives in cancer treatment, they have been broadly investigated as potential drug delivery platforms for intracellular targeting over the years 82. Serda *et al.* fabricated a novel targeted anticancer agent by constructing a kind of highly water-soluble hexakis-glucosamine fullerene derivative for treating pancreatic cancer, which was termed as Sweet-C_60_ and could majorly accumulate in pancreatic stellate cells (PSCs) nucleus 34. Because glucose metabolism-associated energy was necessary for the proliferation and tumorigenesis, the glycoconjugation of fullerene could enhance the cancer-targeting properties to some extent. Confocal microscopy experiments towards PSCs confirmed the outstanding targeting and permeation ability of this synthesized fullerene derivative. In addition, *in vitro* results of phototoxicity studies indicated that Sweet-C_60_ was nontoxic within 1 mg/ml concentrations, and exhibited strong photoactive and photodynamic cytotoxic effect on PSCs under blue and green light, illustrating the great potential of Sweet-C_60_ in PDT.

RNA interference (RNAi) has shown great therapeutic potential in various diseases including cancers due to its ability in silencing certain oncogenes, however, the efficiency of RNAi can be greatly affected due to the lysosomal-mediated degradation 83. To address this issue, Wang *et al.* constructed a kind of amphiphilic fullerene derivative (C_60_-Dex-NH_2_) to deliver siRNA into cancer cells, which not only promoted the lysosomal entrapment, but also cause lysosomal membrane destruction through triggering controllable ROS under visible light irradiation 35. The MDA-MB-231 cells were used to assess the cellular uptake of the constructed nanocomplexes, which showed that their lysosomes became swollen and most nanocomplexes could be observed scattered in their cytoplasm after irradiating with visible light, indicating the successful lysosomal escape of these nanocomplexes. Moreover, a remarkable gene silencing efficiency of this fullerene-based siRNA carrier was verified both *in vitro* and *in vivo*, presenting an innovative approach for siRNA delivery.

Tumorigenesis is often accompanied with metastasis, which is the primary reason of mortality. A variety of proteins that exist in the cytoplasm are associated with tumor metastasis, therefore, these cell metastasis-associated proteins are becoming specific antineoplastic targets in precise cancer treatment 84. Myosin heavy chain 9 (MYH9), a cytoplasm-located protein overexpressed in many cancer cells, involves in the metastasis-related cell motility and epithelial-mesenchymal transition (EMT) regulation process, which is also responsible for undesirable prognosis 85. As functionalized fullerene derivatives display excellent abilities in inhibiting cancer metastasis and survival, among them, aminated fullerene shows much higher cellular uptake in comparison with other fullerene derivatives, therefore, Zhou *et al.* prepared a kind of C_70_ fullerene derivatives (C_70_-EDA) modified with multiple ethylenediamine (EDA) moieties, which was able to inhibit cancer cell migration, modulate intracellular MYH9 distribution, and hinder EMT through binding on MYH9 protein 37. After treating A549 cells with C_70_-EDA, an inhibitory effect on cancer cell migration and EMT process reversion was observed* in vitro*. Therefore, this work by Zhou's group unveiled a novel therapeutic target based on MYH9 and opened a new door to use C_70_-EDA as a promising agent against cancer metastasis.

#### 2.3.2 Fullerene for TME-based anticancer strategies

ECM can not only provide structural and mechanical support for TME components, but also determines cell behavior and phenotype. Dysregulated interactions between ECM and other cell types can lead to serious pathological consequences, such as the occurrence of hepatocellular carcinoma (HCC) 86. Therefore, ECM remodeling is an approach to prevent oncogenesis. A fullerene nanofilm with an artificial ECM-like structure was fabricated by Sosnowska *et al.* via the arc discharge method, which had a high adhesive capacity with cancer cells to change their behavior and reduce their proliferation 36.* In vitro* cell viability and proliferation assessment using HepG2 and C3A liver cancer cells demonstrated that this nanofilm could suppress the proliferation of HCC cells with good biocompatibility. Moreover, other *in vitro* results revealed that this fabricated nanofilm could contribute to the termination of cell cycle of HCC through mechanotransduction, implying that the application of fullerene nanostructures as artificial ECM-like structures could effectively attenuate cancer cell malignancy to improve anticancer therapeutic efficacy. Reprogramming the immunosuppressive TME (ITM) is very important to achieve successful immunotherapy effect. Li *et al*. proposed a strategy by modifying gadofullerene (Gd@C_82_) with β-alanines (GF-Ala) to rebuild ITM, which could induce macrophages to transform from tumor-supportive M2 type to M1 type, ultimately triggering robust antitumor immunity 38. *In vitro* results based on RAW264.7 cells, 4T1 and A549 cancer cells confirmed that both the M1-related cytokines and protein expression were significantly upregulated, and the proliferation of tumor cells showed a remarkable reduction up to 57.9% after GF-Ala treatment, indicating that GF-Ala was able to polarize macrophages to exert an inhibitory tumor growth.

### 2.4 Carbon nanotubes

**Figure 10:**
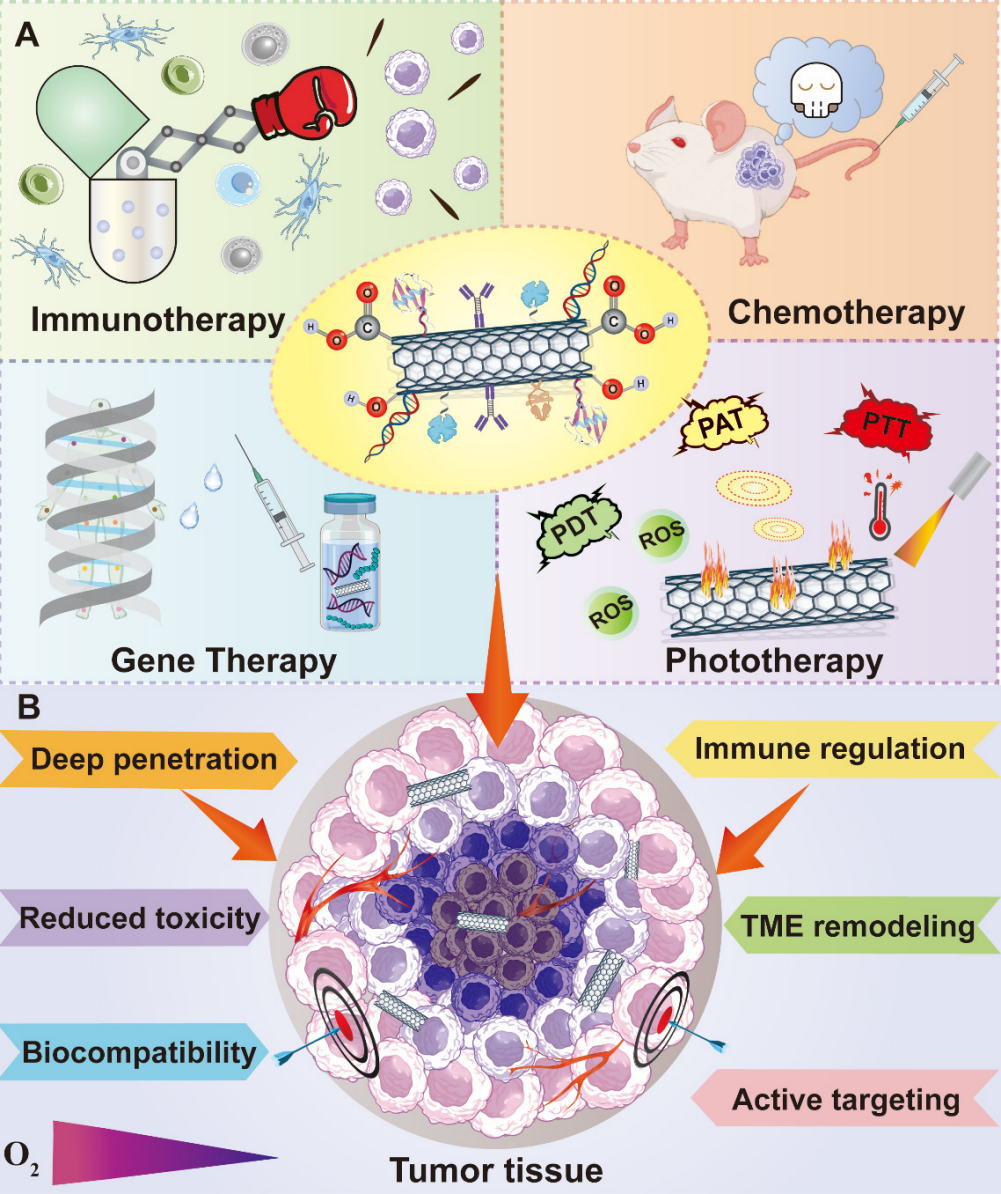
Advantages of CNT functionalization and multiple applications of functionalized CNTs in cancer treatment. CNTs can be applied in many therapeutic fields against cancer including chemotherapy, phototherapy, immunotherapy and gene therapy. Moreover, the modification of CNTs through various functional groups can not only improve their tumor penetration and biocompatibility, but also reduce their innate toxicity and realize active targetability. Abbreviations: PAT, photo-acoustic tomography; PTT, photothermal therapy; PDT, photodynamic therapy; ROS, reactive oxygen species; TME, tumor microenvironment.

CNTs, one of the most widely investigated carbon-based nanostructures, have gained much attention in biomedical fields with multiple application potentials 87. According to the number of the sheet of carbon atoms, CNTs can be generally sorted into single-wall carbon nanotubes (SWNTs) and multi-wall carbon nanotubes (MWNTs), both of which play a key role in cancer treatment and diagnosis 88. Owing to their distinct physiochemical characteristics such as great optical properties, ultra-high surface area for drug loading and functionalization possibilities, CNTs are extensively explored as drug carriers in anticancer targeted delivery 89. CNTs can not only transport various anticancer agents including different chemotherapeutics or biomolecules to intracellular target spots for direct cancer killing effect, but also achieve TME targeting to remodel the microenvironment of cancer cells, which all exhibit excellent anticancer therapeutic effect 90. Though the biological barriers and complex TME often impede the penetration of therapeutic agents into deep tumor sites, due to the needle-like nanostructures of CNTs, they can be easily internalized by many cell types, which enhances their tumor penetration in cancer treatment 91. Besides, the ability of CNTs to absorb light energy in NIR window enables their application in PTT, which facilitates the multifunctional roles of CNTs to serve as both drug vectors and photothermal agents 92. More interestingly, it was reported that CNTs can transform laser energy into acoustic signals and show great photoluminescence as well as Raman scattering in NIR regions, making them excellent candidates in cancer imaging 93. Nevertheless, there are still some limitations that hinder the comprehensive application of CNTs. For instance, CNTs are inherently insoluble with toxicity and can easily get agglomerated, therefore, it is essential to improve their initial properties through surface modification, which can achieve better solubility, enhanced biocompatibility and reduced cytotoxicity in biological systems 94. Recently, many modification methods have been developed to functionalize CNTs either in covalent approaches such as oxidation and carboxylation, or in non-covalent alternatives like π-π bonding or Van der Waals interactions, both largely improving the disadvantageous properties of CNTs 95. Taken together, CNTs are very important carbon-related nanomaterials and contribute a lot to cancer theranostics (**Figure 10**).

#### 2.4.1 Carbon nanotubes for intracellular target-based anticancer strategies

The combinational strategy of gene therapy with PTT was reported to result in better treatment effect than single modality 96, 97. However, the controlled release of therapeutic genes from their vectors is still a challenge that hampers the antitumor efficacy of gene therapy. Zhao *et al.* reported a gene delivery approach using CNTs as carriers to combine with synergistic PTT 39. Briefly, SWNTs and MWNTs were coated with peptide lipid (PL) and sucrose laurate (SL) to construct gene delivery systems that loaded anti-survivin siRNA, which showed outstanding PTT effect and temperature sensitivity. As PL and SL exhibited great sensitivity towards temperature change, while CNTs displayed good photothermal performance upon NIR irradiation, therefore, the constructed gene carriers were photoswitchable and could disassemble after laser irradiation, which facilitated the intracellular release of siRNA and prevented them from endosome trap. The significant antitumor efficiency of the constructed gene delivery vectors was demonstrated *in vitro* and *in vivo*, which could be attributed to the combinational effect of gene therapy and PTT. Notably, some tumors even totally disappeared after treatment with SWNT-PL-carried siRNA for 21 days, indicating the high gene transport capacity of CNTs-based vectors to achieve controlled gene release inside tumor cells. In addition, both SWNT- or MWNT-based gene vectors showed negligible cytotoxicity even their concentrations were up to 60 μg/mL, implying their promising translational potential in the future. Insulin-like growth factor receptor (IGFR) was reported to overexpress in pancreatic cancer cells that are very aggressive, therefore IGFR has widely been regarded as an attractive target against pancreatic cancer 98. Liu *et al.* designed a type of water-soluble, biostable and low-toxic SWNT-based nanocomplexes that were linked with CY7 imaging agent and anti-IGF-1R antibody for pancreatic cancer treatment 40. The constructed nanocomplexes could achieve active targeting to guide cytotoxic PTT under the help of IGFR receptors on tumor cells. Therefore, these novel nanocomplexes could not only aggregate into tumor sites via coupled antibody with minimized damage to normal cells caused by PTT, but also suppress the downstream signaling pathway mediated by IGF-1R to result in additional antitumor activity. The PTT efficiency of the constructed nanoprobes was investigated using the orthotopic pancreatic cancer models *in vivo*, which showed notable therapeutic effect with increased body weight and prolonged survival rate than the control groups, confirming the combinational effect resulted from the adequate accumulation of SWNT-based nanocomplexes in tumor tissues and laser treatment could efficiently ablate pancreatic cancer.

Despite the encouraging effect of PTT, their applications were challenging in deep orthotopic tumor with unavoidable thermal damage to surrounding tissues because of the restricted penetrative ability of laser. To overcome the limitation of phototherapy, a novel therapeutic strategy called thermoacoustic therapy (TAT) based on microwave pulse has emerged. TAT adopts microwave pulse to excite thermoelasticity, and then generates a strong thermoacoustic (TA) shockwave with deep penetrative capability 99. Due to the thermoacoustic properties of CNTs, Wen *et al.* designed a fresh antitumor strategy aiming at deep-penetrated tumor based on SWNTs that served as microwave absorbing agents in targeting mitochondria, which effectively converted microwave power into TA shock wave for selective destruction of tumor mitochondria, thus causing tumor cell apoptosis 41. In their study, SWNTs were functionalized with PL-PEG-NH_2_ to target mitochondria and were triggered by external ultrashort microwave. The TAT effect was investigated *in vivo* using H22 orthotopic liver tumor mice to study the antitumor effect of TAT in deep tumors, which showed that 77.5% of tumor cells were killed due to mitochondrial damage-related apoptosis. Furthermore, microwave is able to penetrate deep in biological tissues, which brought the effective treatment for deep tumors. *In vivo* results confirmed the outstanding antitumor effect of TAT in inhibiting tumor growth, indicating the bright future of CNTs as a kind of promising thermoacoustic agents for cancer treatment.

#### 2.4.2 Carbon nanotubes for TME-based anticancer strategies

The failure of many currently adopted breast cancer treatment is partly attributed to the presence of CSCs that are very difficult to be eliminated through the classical approaches 100. Therefore, Faraj *et al.* designed a type of multimodal nanoplatform using SWNTs to realize noninvasive imaging and specific targeting towards breast CSCs 42. SWNTs were functionalized with PEG and coupled with various imaging tracers for noninvasive tracking. Recently, CD44 has drawn an increasing attention because of its role as the surface marker of CSCs 101. Therefore, CD44 antibodies were conjugated with the PEGylated SWNTs to realize active targetability toward breast CSCs. The biodistribution of CD44 antibody-conjugated-SWNTs monitored through MRI, SPECT and NIR fluorescence revealed that an enhanced selective tumor targeting phenomenon could be achieved in MDA-MB-231 tumor-bearing mice. Besides, the results of immunohistochemistry analysis demonstrated that this nanocarrier could distribute in the tumor sites where CD44 receptors are rich, further confirming the elevated targetability of anti-CD44 SWNTs to CSCs. Cancer nanovaccinology has become an emerging field in cancer immunotherapy, however, the immunosuppressive TME often hinders the immune system for effective tumor eradication. Fortunately, the combinational approach to integrate multiple immunotherapeutic agents in one platform, such as the combination of tumor antigens with different immunoadjuvants can overcome this limitation. For instance, Hassan *et al.* harnessed MWNTs as vehicles to co-deliver immunoadjuvants CpG and anti-CD40 Ig, and OVA antigen for enhanced immunotherapy effect 43. Before loading the cargos onto MWNTs, they covalently conjugated OVA and CpG, which remarkably elevated the adjuvanticity mediated by CpG and was verified by the markedly promoted responses of OVA-specific T cells both *in vitro* and in C57BL/6 mice. Afterwards, anti-CD40 Ig was loaded as the second immunoadjuvant to amplify the antitumor immune reactions. Moreover, MWNTs could improve the co-loading ability of OVA, CpG and anti-CD40 Ig, which significantly inhibited the tumor growth and metastasis in OVA-expressing B16F10 melanoma model. Therefore, this study provided an alternative method to co-incorporate multiple immunotherapeutic agents for efficient cancer immunotherapy.

Tumor vasculature targeting or anti-angiogenesis are promising strategies in cancer treatment. In our previous research, a co-delivery platform based on PEI-functionalized MWNTs was fabricated to address angiogenesis for lung cancer treatment 13. Integrin α_ν_β_3_ has been broadly studied as a therapeutic target for anticancer therapy because of its close correlation with angiogenesis. RGD peptides were demonstrated to bind with integrin α_ν_β_3,_ therefore, it can be applied as targeting ligand for anticancer drug delivery 102. Briefly, iRGD peptide and a kind of angiotensin receptor blocker candesartan were connected to PEI-modified MWNTs, followed by assembly with plasmid angiotensin II type 2 receptor (pAT_2_) through electrostatic interaction to generate the final nanocomplexes. The constructed vector successfully delivered candesartan and pAT_2_ into tumor cells, which contributed to significant tumor growth inhibition and neovascularization suppression in A549 lung cancer model, establishing a perspective platform based on the anti-angiogenetic strategy for lung cancer treatment.

### 2.5 Carbon quantum dots

**Figure 11:**
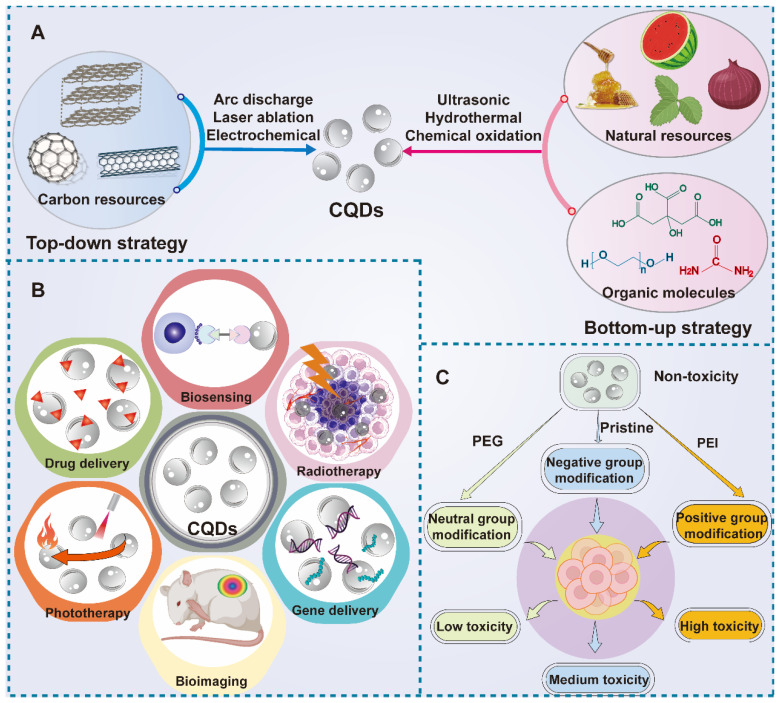
**(A)** Two representative synthetic methods for CQDs including top-down and bottom-down strategy. **(B)** Diverse applications of CQDs in cancer theranostics including drug delivery, bioimaging and biosensing, phototherapy, gene therapy and radiotherapy. **(C)** The toxicity of CQDs after different modification approaches. Abbreviations: CQDs, carbon quantum dots; PEG, polyethylene glycol; PEI, polyethyleneimine.

CQDs are a novel type of carbon-structured 0D materials, which were discovered by Xu's team in 2004 when they prepared SWNTs 103. Because of their good water solubility, biocompatibility, low toxicity, and environmental friendliness, CQDs display a desirable application prospect in many fields 104-107. CQDs can emit light and have good optical stability as well as tunability under illumination. Because of their photoluminescence property, CQDs can be used as electron donors and acceptors. In addition, CQDs can absorb multiple photons simultaneously to cause absorption at shorter wavelengths than the excitation wavelength, which is called upconversion photoluminescence (UCPL) 108. According to the above unique nature, CQDs can be applied in biological imaging, chemical sensors, and biosensors (**Figure 11B**). CQDs can be generally synthesized in two ways: one is "top-down", which indicates the stripping of large-size carbon sources like active carbon, CNTs, carbon fibers and fullerenes into small-size CQDs by electrochemical synthesis, arc discharge and laser ablation 109. The other method is "bottom-up", which means the synthesis of CQDs through carbon materials consisting of small molecules and ions by chemical oxidation, microwave and irradiation (**Figure 11A**). Glucose, urea, and ionic liquids are commonly used as raw materials to synthesize CQDs via this method 108. In particular, the particle size of CQDs can be adjusted in the later stage by controlling different experimental conditions or adopting other methods like ultrasound, and centrifugation 110. Generally speaking, the synthesis method of CQDs is relatively simple with low cost but high yield, which is beneficial to industrial production. Compared to CQDs, the traditional quantum dots are generally extracted from lead or a mixture of silicon and cadmium, which is toxic and environmentally polluting. As a novel class of carbon nanomaterial, CQDs can overcome the shortcomings of traditional quantum dots, which replaces the unstable pairs of fluorescent materials to a certain extent. Besides, due to the wide range of carbon sources and stability, researchers have put forward the definition of green carbon quantum dots (GCQDs) which means the direct extraction of CQDs from vegetables, fruits and other organic materials with low production cost and promising application prospects 111.

The carbon cores of CQDs are non-toxic, however, the modification method and applied dosage will affect their toxicity. For instance, when the concentration of CQDs is higher than 50 μg /mL, it will cause obvious toxicity 112. Among them, the cytotoxicity of CQDs modified by neutral groups such as PEG is the least. In addition, the modification of negative groups such as Pristine will accelerate the process of cell proliferation, on the contrary, the positively charged groups will make the cell cycle stagnant in the G0 phase 113. Other elements such as nitrogen and sulfur can be doped with CQDs to form new hybrid materials, endowing CQDs with other characteristics like photostability and better biocompatibility 114. In addition, surface modification and passivation with functional materials can supplement the functions of CQDs, for instance, passivation with positively charged PEI is able to elevate the affinity between CQDs and cell membrane, which improves their internalization efficiency 115. PEG modification can attenuate the phagocytosis of megakaryocytes and prolong their circulation time *in vivo* (**Figure 11C**) 44. Meanwhile, CQDs can be easily internalized by tumor cells because of their small particle size. Besides, CQDs possess an extensive surface area to load small molecular chemotherapeutic drugs and macromolecular biological drugs, which adds more properties and functions that CQDs do not have. Various tumor-targeting substances and functional NPs can also be combined with CQDs to improve their selectivity and specificity in drug delivery. From this point of view, CQDs are suitable candidates to construct drug carriers for tumor diagnosis and therapy.

#### 2.5.1 Carbon quantum dots for intracellular target-based anticancer strategies

The particle size of CQDs is usually smaller than 10 nm, which facilitates their clearance from the body to limit their application 116. Li *et al.* reported a pH/reduction dual-responsive prodrug micelle composed of hydrophobic acid-labile DOX conjugated CQDs and PEG tail with an average diameter of 127 nm for liver cancer treatment 44.* In vitro* drug release results verified that CQDs-based micelle was able to be cleaved off under the simulated tumor intracellular microenvironment where GSH is overexpressed. Moreover, *in vitro* cellular uptake experiments confirmed that DOX could be released from the carrier and accumulated in the nuclei of cancer cells. *In vitro* cytotoxicity assays also revealed that the proposed micelle could result in more inhibitory effects on HepG2 cell growth than free DOX, indicating the good effect of this nanocarrier in on-demand drug delivery to target tumor parenchyma. Due to the photoluminescence property of CQDs, delivery systems based on CQDs can reflect tumor-related information. In the context of CQDs-based gene therapy, Wang *et al.* adopted positively charged Alkyl-PEI2k to passivate CQDs via electrostatic interactions to load siRNA and pDNA because CQDs and pDNA molecules are both negatively charged, the gene cannot attach to the CQDs tightly if without surface modification 45. *In vitro* transfection experiment proved that the nanocomplex was conducive to promote pDNA delivery into the cytoplasm and then transport it to nucleus for gene expression. In addition, siRNA was also successfully delivered into the breast tumor cells to achieve remarkable gene silencing effect. This study verified that CQDs could be applied as a reliable gene vector for efficient gene therapy.

Multidrug resistance (MDR) is a challenging obstacle that limits the efficacy of chemotherapeutics. Many approaches have been developed to overcome MDR such as inhibition of P-gp overexpression, and D-α-tocopheryl polyethylene glycol succinate (TPGS) was reported to suppress the overexpression of P-gp 117. With this aim in mind, Zhang* et al.* combined mitochondria-targeted triphenylphosphine (TPP) and TPGS to fluorescent CQDs to deliver DOX by self-assembly for resistant breast cancer treatment 46. *In vitro* cell viability assay using DOX-resistant MCF-7 cells demonstrated that the IC_50_ of CQDs nanocomplexes-treated cells was much lower than free DOX, indicating the novel nanocomplexes had better treatment efficiency than free DOX. Moreover, the substantially reduced drug resistance index (RI) reflected that the nanocomplex successfully converting highly DOX resistant cells (MCF-7/ADR) into moderately resistant. Furthermore, the constructed nanocomplex caused significant decreases in mitochondrial membrane potential (ΔΨm), whose numerical decline was a symbol of mitochondria-triggered apoptosis. Notably, the nanocomplexes-treated multicellular tumor spheroids presented not only significant morphological changes but also a remarkably reduced volume, indicated that CQDs-based mitochondrial-targeted drug delivery system could inhibit MDR development and provided new ideas for antitumor therapy.

#### 2.5.2 Carbon quantum dots for TME-based anticancer strategies

MMPs are a class of enzymes overexpressed in TME that have the ability to decompose ECM, reshape tumor basement membranes, accelerate angiogenesis and promote the process of tumorigenesis. Cu-metal-organic framework (MOF) materials are capable of destroying the structural integrity of F-actin which is the cytoskeleton component of ovarian cancer cells, thus inducing tumor cell necrosis 118. Chen* et al.* firstly fabricated TME targeted CQDs/Cu_2_O nanocomplexes for ovarian cancer treatment 47. A series of *in vitro* experiments showed that CQDs/Cu_2_O could not only destroy the structural integrity of F-actin but also cause downregulatory expression of MMP-2/9 and VEGFR2, which greatly hindered angiogenesis in TME. Furthermore, the novel nanocomplex could regulate the expression of multiple genes in cancer cells to effectively inhibit their growth and migration. Suppressor of tumorigenicity 14 (st14) is a type II transmembrane serine protease that is specifically expressed on the tumor surface, reducing the expression of st14 gene or preventing proteolysis activity can significantly inhibit the proliferation and metastasis of tumor cells 119. Kunitz domain 1 (KD1) is an efficient potent st14 inhibitor, however, the short residence time *in vivo* greatly restricts the scope of its application 120. In order to reduce the renal clearance rate of KD1, Hu *et al.* combined CQDs with KD1 for breast cancer treatment. On one hand, the nanocomplexes could specifically reach the microenvironment of tumor sites. On the other hand, they could effectively inhibit the activity of st14 48. The results of *in vitro* imaging experiments indicated that CQDs-KD1 distributed around tumor cells and took effect in TME. Besides, *in vitro* invasion results proved that CQDs-KD1 could suppress the invasion of MCF-7 cancer cells by reducing the degradation of base membranes (BMs) and ECM. Moreover,* in vivo* experimental results marked that the accumulation of CQDs-KD1 in tumor tissue was about 3.6 times higher than that of free CQDs, which resulted in a smaller tumor size and weight with significant lung metastasis suppression compared to the control group.

## 3. Carbon nanomaterials for cancer diagnosis

**Figure 12:**
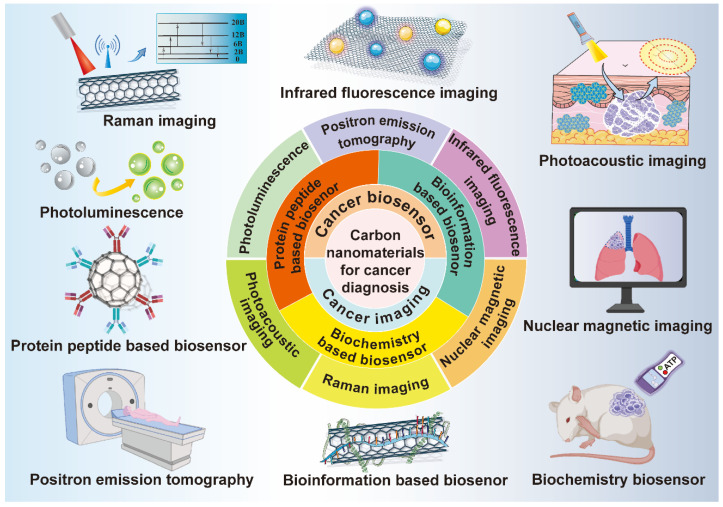
Schematic illustration of various carbon nanomaterials in cancer diagnosis. Carbon nanomaterials play critical roles in the field of cancer diagnosis, which are widely used in different kinds of cancer imaging methods and biosensor.

Diagnostic technology is crucial for the detection and prediction of multiple cancers. The early diagnosis and intervention for cancer patients can not only prolong their survival rate, but also improve their life quality, therefore, cancer diagnostic technology is particularly significant in cancer treatment. However, the commonly adopted diagnostic methods have the shortcomings of adverse effects, low security, high cost, poor sensitivity and bad targeting effect, thus, developing and updating the existing cancer diagnostic approaches are of great necessity 121. Most carbon nanomaterials possess good biocompatibility, extensive sources and simple preparation methods, so they show great potential to be applied in cancer diagnosis 50. In this section, different diagnostic approaches including lots of cancer imaging methods and biosensors based on various carbon-based nanosystems will be discussed (**Figure 12**).

### 3.1 Cancer imaging

Cancer imaging plays an important role in cancer diagnosis. There are many imaging technologies such as computed tomography (CT), fluorescence imaging, MRI, PAI, single-photon-emission computed tomography (SPECT), ultrasonography (US), and positron emission tomography (PET) and Raman spectra, all of which constitute the crucial part of cancer imaging 122. CT is mainly based on the absorption of X-ray that has multiple absorption coefficients when penetrating diverse organs or tissues of different compositions and densities to generate corresponding pixel values. Finally, CT images are formed by converting CT values of different pixels to gray scales 123. US belongs to a low-cost and easy-to-operate imaging method that uses ultrasonic beams to scan the human body and obtain images of internal organs and tissues by receiving and processing reflected signals 124. MRI is a widely used imaging tool without ionizing radiation damage to the human body and with good resolution to various tissues, which can form original 3D cross-section images 125. PET can be carried out by injecting glucose, protein, nucleic acid and other bioessential substances that are marked with short-lived radionuclides into the human body. Then, the aggregation of these substances in the body is observed to reflect the situation of life metabolic activities to achieve diagnostic purpose. PET is widely applied in tumor diagnosis in clinic because of its high sensitivity and specificity 126. Moreover, PET can be combined with CT or MRI to achieve better accuracy and positioning 127. PAI is a novel non-invasive and non-ionized biomedical imaging method. When the pulsed laser irradiates the biological tissues, ultrasonic signals will be generated to reflect the light absorption characteristics of the tissue and form image. PAI has the advantages of high selection and deep penetration, which can obtain high resolution and contrast tissue images 128.

The majority of carbon nanomaterials possess extensive infrared absorption, excellent fluorescence properties and obviously intrinsic Raman vibration signals, therefore, they are able to be utilized as effective tools for cancer imaging 50. Meanwhile, carbon-related nanomaterials have a large surface area with good modifiable ability, so they can also be utilized to deliver various contrast agents or imaging agents for cellular tracking. The following paragraphs focus on the different imaging approaches based on various carbon nanosystems, which highlights the advantages of these carbon structures and emphasizes their application perspectives in cancer diagnosis. **Table 2** is the summary of some representative applications of carbon-based nanosystems in cancer imaging.

#### 3.1.1 Graphene-based nanosystems

Graphene and its derivatives have gained plenty of attention in cancer imaging because of their unique physiochemical properties. For instance, graphene and its oxide possess strong fluorescence quenching ability, while graphene quantum dots (GQDs) have photoluminescence characteristics, which are closely related to their preparation methods and morphological features 149. Moreover, the application of graphene-based nanomaterials can attenuate the several adverse effects during cancer diagnosis because some of the currently adopted contrast agents like quantum dots are toxic, which would cause several side effects to a part of cancer patients who have already undergone organ damages due to chemotherapy 150. In addition, graphene-based nanomaterials have outstanding photostability, which enables their application in many biological imaging like PAI, MRI, PET, US and CT 50.

Yang *et al.* labeled two Cy5 molecules on the relative ends of a stem-loop molecular beacon (MB) that can specifically identify nucleic acid, and their fluorescence signals would decrease due to the self-quenching effect 151. Then, MB was bound to the GO surface and the intermolecular fluorescence quenching of Cy5 was further enhanced by the action of fluorescence resonance energy transfer (FRET). When the nanocomplex entered into the tumor cell and bound to the target molecule miRNA-21, the combination was destroyed and the fluorescence signal of Cy5 was restored. This method reduced the fluorescence background of the imaging and significantly improved the fluorescence intensity. As *in vitro* and *in vivo* results demonstrated, the constructed nanocomplexes had great imaging ability for multiple tumor cells, indicating that GO-based nanomaterials had promising potential in cancer diagnosis. Moreover, GO and rGO are also considered as outstanding PA reagents. Hu* et al.* prepared a nanosystem based on polydopamine (PDA)-rGO and connected the diagnostic reagent ICG to it for breast tumor PTT and PAI 152. PDA was able to enhance the water solubility and biocompatibility of the nanosystem, quench the fluorescence of rGO and enhance the PAI efficiency. *In vivo* imaging experiments showed that the ICG-PDA-rGO processed higher sensitivity and PAI ability than other control groups. Meanwhile, the vascular tissue could also be clearly observed in tumor tissue because of the endogenous contrast agent hemoglobin. The above observations indicated that rGO-based nanomaterials could not only be used in PTT to improve the efficiency of cancer treatment but also could be applied in PAI for tumor diagnosis.

Liu *et al.* reported 9T-GQDs with strong absorption in the NIR-II region by adjusting the decomposition of H_2_O_2_ and using phenol as the precursor in a 9T magnetic field for breast, cervical and lung cancer imaging 153. The upconverted photoluminescence emission of 9T-GQDs could be excited by the long-wavelength light, and the PL quantum yield of the 9T-GQDs was 16.67%, which was almost 1.8 times more than ordinary GQDs. Interestingly, the 9T-GQDs could penetrate the 4T1 cancer cell membrane and enter into the cytoplasm to induce continuous fluorescence. Su *et al.* reported a dual imaging nanoprobe based on GQDs that attached to core/shell structure Fe_3_O_4_@SiO_2_ and loaded DOX for MRI imaging of cancer cells 154. Interestingly, GQDs could produce blue fluorescence under the UV light excitation of 365 nm, and when it connected with the red fluorescence of DOX, the whole fluorescence was quenched due to the formation of the FRET system. When the GQDs-based nanocomplex was ingested by the tumor cells, the FRET system was destroyed and the DOX was released. In addition, Fe_3_O_4_ could enhance the negative image contrast of Hela cells which was conducive to MRI, illustrating the potential of GQDs-based nanosystem for cancer diagnosis.

#### 3.1.2 Fullerene-based nanosystems

Recently, fullerene and its derivatives are used as effective tool for cancer imaging because of their optical features such as excellent fluorescence properties. Kwag *et al.* synthesized a kind of hyaluronated fullerenes with strong NIR fluorescence intensity, which enabled the *in vivo* florescent imaging of tumor sites with high-resolution 137. The generated hyaluronated fullerenes showed great water solubility, photosensitivity, specificity tumor targeting, and robust fluorescent signal without conjugating with other fluorophores or isotopes, which achieved the non-invasive photoluminescent imaging of KB and HCT-116 tumor-bearing mice. Shi *et al.* reported a PAI-guided strategy for cancer diagnosis based on fullerene 79. They conjugated D-A antenna onto the fullerene to red-shift their main absorption into the NIR window, followed by DSPE-mPEG coating to enhance the biocompatibility of the whole nanocomplexes. *In vivo* results obtained from HeLa cell-bearing mice showed that the constructed nanocomplexes could produce a strong PA signal for biological imaging, which guided the synergistic PDT and PTT for efficient cancer elimination.

#### 3.1.3 Carbon nanotube-based nanosystems

As a class of inorganic nanomaterials with many distinct features, CNTs have gained much interest in cancer diagnosis. CNTs can be applied in various cancer imaging techniques like MRI, PAI, Raman imaging, ultrasonography, radionuclide imaging and NIR fluorescence imaging 155. For instance, Lovell *et al.* reported a sort of CNTs-based nanohoop that could generate fluorescence for *in vivo* cervical carcinoma cell imaging 156. Coating noble metals around the surface of CNTs is often used to improve their Raman signals 157. Wang *et al.* functionalized PEGylated CNTs with gold or silver to realize the surface enhanced Raman scattering (SERS) effect of constructed nanocomposites 138. Upon NIR irradiation, CNTs modified with noble metals required the least time to obtain the Raman images in comparison to single CNTs. MRI belongs to a kind of tomography that utilizes magnetic resonance phenomena to obtain electromagnetic signals from the body, which finally displays the relevant information for tumor detection. Zhang *et al.* functionalized MWNTs with gadolinium NPs (GdN), magnetofluorescent CQDs and FA for dual-modal targeted cancer imaging 143. The modifications of MWNTs improved the stability and water solubility of MWNTs, which were essential for their applications in MRI.* In vitro* results showed that the nanocomplexes could amplify the longitudinal proton relaxation process, which testified the feasibility of these nanocomplexes to be used as T1 contrast agents in MRI.

#### 3.1.4 Carbon quantum dot-based nanosystems

CQDs are a kind of novel fluorescent nanomaterials with excellent biocompatibility, optical characteristics and accessible synthesis methods, making them a rising star with wide application perspectives in cancer diagnosis. Nevertheless, bare CQDs are often weakly fluorescent, therefore, engineering CQDs to endow them with desirable labeling and imaging properties is of great significance 158. For instance, Shen *et al.* synthesized a type of amine-rich CQDs with blue fluorescence, which was conjugated with tumor nucleolar targeting aptamer AS141 and connected to the NIR fluorescence group Ce6 through a substrate that was sensitive to Cathepsin B overexpressed in the lysosome 147. Due to the FRET between CQDs and Ce6, the light of CQDs was quenched at 450 nm, while the fluorescence of Ce6 at 650 nm was significantly enhanced. When the nanocomplexes reached to the tumor site, the FRET process was terminated because of the dissociation of Ce6, resulting in the significant emission wavelength shift from the NIR region to the blue region, which exhibited a typical dual-emission fluorescence image for detecting cervical carcinoma cells. In addition, this CQDs-based nanocomplexes have the advantages of low detection limit, high sensitivity, which is beneficial to their clinical application. Li *et al.* designed an approach for long-term Golgi imaging using _L_-cysteine modified CQDs (LC-CQDs) 148. The prepared LC-CQDs exhibited great fluorescence, high quantum yield with 68%, good photostability and biocompatibility. The long-term imaging capabilities of Golgi was determined using laryngeal Hep-2 cancer cells, which offered clear and real-time images to monitor the Golgi apparatus. This study provided an innovative approach to diagnostically investigate the intracellular morphological change of Golgi, which is valuable for the future cancer diagnostic methods based on subcellular organelle.

### 3.2 Cancer biosensor

Biosensor usually refers to the device that combines bio-sensitive components with converters to detect certain bioactive or chemical substances with good specificity and selectivity 159. The measured substance can be analyzed quickly and accurately from a molecular point of view. Up to now, there are many types of biosensors, among which electrochemical biosensors are the most widely used and relatively mature technologies 160. In general, there are many biomarkers in tumors, which can be roughly divided into three categories. The first category is the biological information of the tumor cell itself, such as DNA and mRNA. The second type is the biomolecular or chemical signal in TME, like ATP and GSH. The last one contains proteins and enzymes expressed in tumor cells or TME, such as carcinoembryonic antigen (CEA) and alpha-fetoprotein (AFP) 161. Some carbon nanomaterials can be used as signal converters to transfer chemical or physical changes in organisms into available signals or serve as an enhancer to strengthen the signals 162. In addition, carbon-based nanomaterials have the advantages of simple operation, low detection limit, fast detection time, and strong selectivity, which are all suitable for application in cancer biosensors. However, it has to be admitted that the complex preparation process, short service life, and high cost may be the key factors that restrict their clinical transformation.

**Figure 13:**
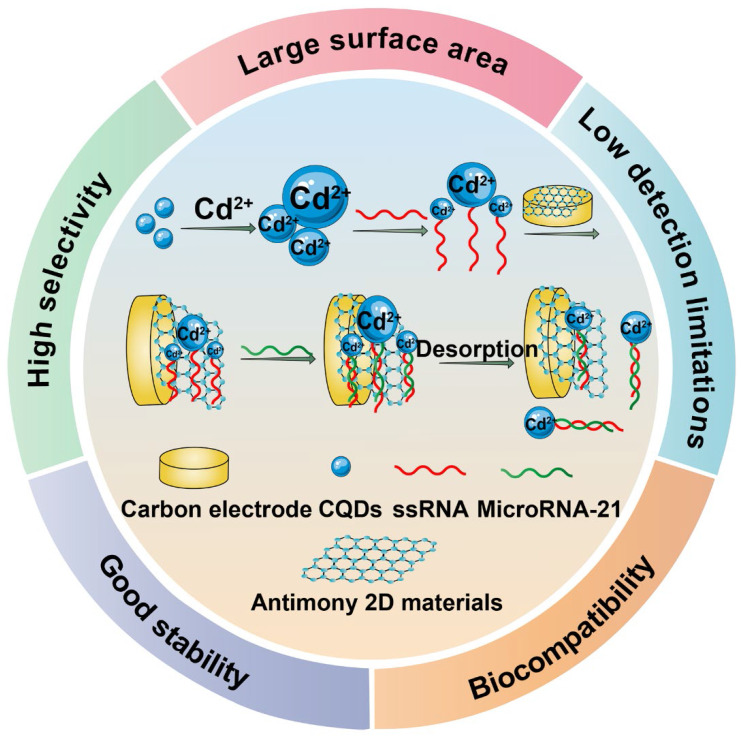
Schematic diagram of the construction of miRNA biosensor and its detection principle and advantages such as high selectivity, good stability, biocompatibility and low detection limitation. Abbreviations: CQDs, carbon quantum dots; ssRNA, single-stranded RNA; 2D, two-dimensional.

Cancer antigen 125 (CA 125) is a kind of mucin-like glycoprotein overexpressed in ovarian tumor cells, so it can be considered as a biomolecule marker antigen for the detection of ovarian cancer 163. Pakchin *et al.* designed a novel biosensor by modifying 3D rGO-MWNTs on the carbon electrode to improve their conductivity and increase their specific surface area, then they connected the carbon electrode to polyamidoamine-linked gold NPs to further increase their conductivity and the number of antibody loads 164. Finally, chitosan (CS) was covered on the surface to improve the solubility of the electrode. *In vitro* performance analysis experiment verified that the linear range of the immunosensor is wide, and the detection limit was as low as 6 μU/mL. Similarly, Biswas *et al.* covered the carbon electrode with CNTs to connect them with a kind of MOF material Zr-trimesic acid, which displayed excellent electrocatalytic ability and protein compatibility to detect CA 125 antigen 165. The expression level and environment of miRNA can reflect biological information, which is able to serve as biomarkers to evaluate the status of cancers and predict their development 166. Zhou *et al.* designed an ultrasensitive electrochemical based on thiol group multi-labeled C_60_ NPs for miRNA-141 detection 167. First of all, they linked two cleavage sequences containing G-quadruplex via chemically mediated nucleic acid chains, the yield G-quadruplex was complementary to miRNA-141 to form a DNA-RNA hybrid double strand. Then, the DNA double-strand was cleaved and broken by duplex-specific nuclease to release miRNA, and the original signal was amplified by enzyme-assisted target recycling. Compared with a single amplification strategy, the novel electrochemical biosensor based on C_60_ exhibited more excellent recognition ability and better analysis efficiency. In addition, Zhang *et al.* developed a novel electrochemical biosensor based on CQDs 168. The antimonene nano-flakes and CQDs were used as the substrate of cadmium ion for specific detection of miRNA-21 which is a biomarker associated with breast cancer. After the complementary miRNA was added, it was easy for the hybridized target to desorb from the antimonene interface because of the low adsorption affinity of dsRNA to antimonene, meanwhile, the oxidation peak of metal ions could be remarkably decreased. As the experimental results demonstrated, this biosensor could detect the miRNA-21 concentration ranging from 100 aM to 1 nM with its detection limit of as low as 21 aM, which was three times lower than established miRNA biosensors (**Figure 13**).

## 4. Clinical translatability and challenges

The clinical translatability of carbon nanomaterials is an issue that cannot be avoided and many challenges remain to be overcome before their successful translation in practice. Although carbon nanomaterials can serve as effective tools with outstanding properties in cancer theranostics, bridging the gap between laboratory research and clinical transformation still needs a long way to go. The biodegradability and metabolism of carbon nanomaterials are important concerns that hinder their clinical application. For graphene and derivatives, their toxicity is a major concern that should be taken into consideration for biomedical application in clinic. The cytotoxicity of these nanomaterials is highly dependent on their inherent flake sizes, their structure and shape, surface chemistry, and the type of cultured cells 169. Therefore, more studies can be carried out to investigate ideal graphene-based nanomaterials with low cytotoxicity by controlling their size and suitable decoration. Besides, to accelerate their clinical translation, many problems should be resolved in advance, including their stability in physiological conditions, cellular uptake, biodistribution and accumulation in different tissues and organs, transformation and metabolic pathway *in vivo*, acute and latent toxicity 170. In addition, the delivery efficiency of therapeutic agents into desired region play a vital role in obtaining expectant theranostic goals. The suitable size of graphene-based nanomaterials is a key point for EPR-mediated passive tumor targeting 171. Moreover, the over-expressed receptors on tumor cytomembrane should be explored to achieve active targeting. Meanwhile, after successful delivery of graphene-based nanoplatform into tumor tissue, the endogenous or exogenous stimuli should be fully utilized to achieve controlled drug release. Although graphene and derivatives can be tailed as versatile platform to achieve multiple theranostic purposes because of their inherent advantages, in present strategies, many challenges still exist in getting such multifunctional platform, including complicated designing, tedious synthesis, inadequate synergistic functions, low integrated efficiency, and uncertain biological response. Therefore, plenty of attention should be paid to fabricating such platform with combinational functions in a rational way.

The anti-tumor properties of fullerene derivatives have attracted many researchers' attention worldwide 80. However, their antineoplastic mechanism has not been thoroughly clarified and there is a lack of understanding regarding their regulation mechanism of tumor proliferation and metastasis. Before their direct application as anti-tumor drugs in clinic, more work needs to be devoted to determining the metabolic pathway and biological safety of fullerene derivatives in the body and how they themselves can be used as anti-tumor agents for cancer treatment, which might be helpful to overcome the limitations of conventional anti-tumor drugs. Moreover, because fullerene-based structures possess abundant cyclohexanes, they are very aromatic and have stable and inert carbon bonds. Thus, their insolubility in aqueous media and poor miscibility limit their biological applications to a great extent, and their strong tendency to form self-aggregate also leads to phase separation problems 172.

For CNT-mediated drug delivery, free CNTs can be retained in the body after drug dissociation, which causes secondary damage 173. Moreover, the mechanisms of action of CNTs on normal cells and tissues are not fully understood, thus impeding their clinical application. Due to the special structure of CNTs, potential toxic effects can occur. Therefore, more efforts should be made to explore suitable functional molecules for the modification of CNTs, which improves the biocompatibility to promote their translatability in clinic. In addition, clarifying the mechanisms of CNT-induced toxicity is also an approach which might be beneficial to avoid the toxic and risk factors in designing CNT-based nanoplatform. Moreover, the biodegradability of raw CNTs are low because the hydrophobic interaction prevents enzymes from approaching it, however, the functionalization of CNTs can enhance their solubility and create defect sites, which offer desired binding sites for enzymes and promote their enzymatic degradation 174. Similar like the aforementioned carbonaceous nanomaterials, GDY is also facing the challenge in how to transform from experimental findings to clinical practice. Although several studies indicated that GDY and derivatives were more biocompatible than other carbon-based nanomaterials, the long-term biotoxicity of these nanomaterials are still not clear 175, 176. Like other 2D carbon nanomaterials, the accumulation of GDY in different tissues and their excretion time have an impact on its long-term biotoxicity 177. Thus, systematic evaluation on the biotoxicity and metabolism of GDY-based nanomaterials are of urgent necessity. Moreover, the mass production of homogeneous GDY or other carbon nanomaterials is the precondition for their clinical application, therefore, developing reliable preparation method that is suitable for large-scale production and can maintain stable physiochemical characteristics and morphology plays a fundamental role in their clinical translation.

CQDs are widely used as fluorescent nanomaterials in various biomedical fields due to their good biocompatibility, multifunctionality, adjustability and designability 178. The wide range of raw material sources for CQDs makes them unique among other carbon nanomaterials. Particularly, the synthesis of CQDs using therapeutic drugs as raw materials show promising perspective in biomedical application. The drug-derived CQDs can exhibit the photoluminescence features of ordinary CQDs and retain the good pharmacological effects of therapeutic agents 179. However, it is still a daunting challenge to produce CQDs with well-defined structures and morphologies, which requires the suitable selection of raw materials, reaction conditions and synthesis approach. Currently, many mechanisms for photoluminescence are still in the hypothesis stage, and how to effectively synthesize controllable CQDs with photoluminescence has become a major problem in their clinical translation. Most CQDs are reported to exhibit blue or green emission, only a few of them display red fluorescence 178. However, red/NIR emission shows better imaging capability in deep tissue due to its strong penetrability 180. Therefore, more research can be investigated to explore the controlled synthesis of red/NIR CQDs with high emission efficiency. Hybrid CQDs with long-lived luminescence is another research orientation that is beneficial to their biomedical applications because these kinds of CQDs are able to avoid the significant interference from autofluorescence 181. In addition, the design and fabrication of CQD-based nanoprobe for specific organelle imaging is another promising research focus in the future. To sum up, the unique physiochemical characteristics of CQDs make them attractive for a number of scientific themes. With the aim to facilitate their better clinical translation, appropriate synthesis methods, accurate structure analysis, as well as the deep understanding of their biological behaviors at the molecular level and their synthesis mechanisms still need further exploration and research.

Encouragingly, compared with carbon nanomaterials, another kind of inorganic nanomaterials, silica nanoparticles (SiNPs), have already achieved success in their clinical practice. Bradbury and teammates reported the first-in-human clinical trial of ultrasmall hybrid SiNPs labeled by ^124^I which were termed as Cornell dots for the PET imaging of patients with metastatic melanoma, instilling more confidence in the clinical transformation of radiolabeled inorganic NPs for cancer diagnosis 182. Since then, ultrasmall core-shell SiNPs have been widely investigated for the precise imaging and delivery of therapeutic agents for multiple cancer theranostics, including high-grade malignant brain cancer, prostate cancer and lung cancer, revealing the great clinical potential of these inorganic NPs 183-186. Although there is no clinical practice that have ever reported the application of carbon nanomaterials as drug delivery vehicles in cancer theranostics, there are several cases that reported the utilization of carbon nanoparticles (CNPs) in lymphatic mapping during colorectal cancer surgeries, lymph nodes harvest in advanced gastric cancer, and lymph node biopsy of papillary thyroid carcinoma, illustrating the tremendous potential and promising future of carbon nanomaterials in cancer theranostics 187-189.

## 5. Conclusion

This review provides an overview of the update achievements of carbon nanomaterials in cancer treatment and diagnosis using substantial examples and cases. There is no doubt that the flourished development of nanotechnology has contributed a lot to biomedical fields. From all the nanomaterials, carbon-related nanostructures have shown tremendous advantages in cancer theranostics due to their distinctive physiochemical properties. Their extensive surface area can be conjugated with many therapeutic agents, imaging molecules and targeting moieties, which significantly improves their targetability, specificity and therapeutic performance. Because of their natural optical features, thermal ablation of tumor cells can be realized through NIR irradiation. Moreover, multifunctional platform that integrate diagnostic and therapeutic functions can be simultaneously achieved because of their versatility, which largely benefits the development of a number of novel theranostic modalities. Of course, in addition to the aforementioned carbon nanostructures, other carbonaceous nanoplatforms like carbon nanohorn and porous carbon all show tremendous potential against various cancer types 190.

Despite the inspired advancements in this fascinating field, issues and challenges still exist, which requires more efforts to be dedicated in addressing them. For instance, their long-term nanotoxicity and pharmacokinetics should be carefully evaluated using more suitable animal models because these properties are highly dependent on many parameters such as the structure, synthesis method, antitumor mechanism, dosage and exposure time. It is also necessary to study the impact of carbon nanostructures on immune system, central nervous system and reproductive system, however, excluding these influence needs more thorough and careful investigation. Besides, carbon-related nanomaterials are usually non-uniform, which causes difficulties in standardizing the preparation and functionalization methods of these nanomaterials, so there is still some space for improvement to establish more stable and repeatable approaches in constructing carbon-based nanoplatforms 191. In addition, although phototherapy has shown great perspective in cancer treatment, the precise delivery of local heat to tumor cells for efficient tumor killing effect is still challenging. Therefore, more work should be done to achieve synergistic phototherapeutic performance through combining the optical property of carbon carriers and photoactive agents. In the context of cancer diagnosis, it is always a tricky problem that some tumors are too small to be detected in their early stage, therefore, it is urgent to explore novel diagnostic approaches based on carbon nanomaterials with good accuracy and high efficacy to detect very tiny tumors, which is helpful for their earlier identification to seek for the optimal treatment modality.

However, opportunities and challenges always coexist. Solving each of these aforementioned obstacles will add more chances in developing novel theranostic tools based on carbon nanomaterials. With the rapid progress in scientific technology, it can be expected in the future that these challenges can be overcome and carbon nanomaterials will show excellent performance in the arena of cancer theranostics.

## Figures and Tables

**Table 1:** Summary of carbon nanomaterials in cancer treatment

Carbon nanomaterial	Therapeutic target/modulation	Drug delivery system	Therapeutic modality	Tumor therapy model	Effectiveness	Ref.
GO	Mitochondria	PTX@GO-PEG-OSA	PDT, PDT and Chemotherapy	HGC-27 gastric cancer	Induce mitochondrial damage, suppress the energy supply of P-gp, reverse the drug resistance of PTX	24
	Cytoplasm	GO-PEI-miR-214 inhibitor	Gene therapy	Cal27 and SCC9 oral squamous cell carcinoma	Decrease OSCC cell invasion and migration, increase cell apoptosis by targeting PTEN and p53	25
	Nucleus	C-dot-PEG-pDNA-TNF-α-CS-CGO	Gene therapy	Hela cervical cancer	Actively target tumor cells and deliver pDNA into the nucleus, generate anti-angiogenesis effect	26
	Immune cells	GO-PEI-R848-mRNA	Gene therapy and Immunotherapy	B16 melanoma	Generate OVA-specific antibodies, decrease the tumor size and weight, prevent the lung metastasis	27
	Redox /pH/enzymatic responsive drug release	GGBD	PTT and Chemotherapy	MCF-7 breast cancer	Release DOX in TME, improve the drug penetration ability and enhance intracellular delivery of drug	28
GNR	Extracellular matrix	GNR-PEG-Ce6	PAT	SKOV-3 ovarian cancer	Delay the disaggregation and spreading of ovarian cancer spheroids, reduce their adhesion to ECM protiens	29
GDY	Cytoplasm	GDY-CeO_2_-miR181a	Gene therapy	Esophageal squamous cell carcinoma	Facilitate DNA damage, relieve hypoxic tumor environment, and sensitize radiotherapy	30
GDYO	Nucleus	GDYO-CDDP/DOX@DSPE-PEG-MTX	PTT, PDT and Chemotherapy	Hela cervical carcinoma	Enhance active target ability and achieve excellent synergistic photo-chemotherapy effect	31
	ROS modulation	rGDYO-Fe_3_O_4_-CREKA	PTT and Fenton reaction-mediated therapy	4T1 breast cancer	Achieve synergistic PTT and Fenton reaction-mediated antitumor effect	32
	Tumor vasculature/Hypoxia	GDYO@i-RBM	PTT and PDT	EMT-6 breast cancer	Alleviate tumor hypoxia, improve blood perfusion, and achieve synergistic PDT and PTT effect	33
C_60_	Nucleus	Hexakis-glucosamine C_60_ (sweet-C_60_)	PDT	Pancreatic cancer	Enhance tumor targeting and exhibit strong photoactive and photodynamic cytotoxic effect	34
	Lysosomal	C_60_-Dex-NH_2_	Gene therapy	MDA-MB-231 breast cancer	Promote the lysosomal entrapment of siRNA and exhibit remarkable gene silencing efficiency	35
	Extracellular matrix	C_60_ nanofilms	Chemotherapy	HepG2 and C3A liver cancer	Suppress the proliferation of HCC cells and terminate their cell cycle	36
C_70_	Cytoplasm	C_70_-EDA	Chemotherapy	A549 lung cancer	Inhibit cancer cell migration, modulate intracellular MYH9 distribution, and hinder EMT process	37
Gd@C_82_	Macrophages	β-alanines modified Gd@C_82_ NPs	Immunotherapy	4T1 breast cancer model and A549 lung cancer	Upregulate M1-related cytokines and protein expression, reduce the proliferation of tumor cells	38
SWNTs	Cytoplasm	SWNT-PS/siRNA	PTT and Gene therapy	Hela cervical cancer	Generate high gene transport capacity and achieve controlled gene release in tumor cells, exhibit high antitumor activity	39
	Cytoplasm	SWNT-CY7-IGF-1Ra	PTT and Immunotherapy	Pancreatic cancer (ASPC-1, BXPC-3, PANC-1, and SW1990)	Achieve precise tumor-targeting therapy, increase the body weight and prolong the survival rate of tumor-bearing mice	40
	Mitochondria	SWNTs-PL-PEG-NH2	TAT	H22 liver cancer	Selectively destruct tumor mitochondria, cause tumor cell apoptosis	41
	Cancer stem cells	/	Immunotherapy	MDA-MB-231 breast cancer	Realize active targetability toward breast CSCs	42
MWNTs	Antigen-presenting cells	MWNTs-CpG-αCD40-OVA	Immunotherapy	B16F10 melanoma	Improve the co-loading ability of OVA, CpG and anti-CD40 Ig, inhibit tumor growth and metastasis	43
	Tumor vasculature	iRGD-PEI-MWNT-SS-CD/pAT_2_	Chemotherapy	A549 lung cancer	Result in significant tumor growth inhibition and neovascularization suppression	13
CQDs	Nucleus	PEGylated CQD-DOX	Chemotherapy	HepG2 liver cancer	Accumulate in the nuclei of cancer cells and inhibit HepG2 cell growth	44
	Cytoplasm	Alkyl-PEI2k-Cdot	Gene therapy	4T1 breast cancer	Promote pDNA delivery into the cytoplasm and achieve remarkable gene silencing effect	45
	Mitochondria	CQDs-TPGS-TPP	Chemotherapy	MCF-7 breast cancer	Inhibit MDR development and trigger tumor cell apoptosis	46
	Tumor vasculature	CQDs/Cu_2_O nanocomplexes	Chemotherapy	SKOV3 ovarian cancer	Hinder angiogenesis in TME and inhibit tumor cell growth and migration	47
	Extracellular matrix	CQDs-KD1	Chemotherapy	MCF-7 and 4T1 breast cancer	Prolong retention time of KD1 in plasma and at the tumor site, effectively inhibit tumor growth and lung metastasis	48

Abbreviations: CD40, clusters of differentiation 40; CDDP, cisplatin; C-dot, carbon dot; Ce6, chlorin e6; CpG, cytidine-phosphate-guanosine; CQD, carbon quantum dot; CSCs, cancer stem cells; Dex, dextran; DOX, doxorubicin; ECM, extracellular matrix; EDA, ethylenediamine; Gd, gadolinium; GDY, graphdiyne; GDYO, graphdiyne oxide; GNR, graphene nanoribbon; GO, graphene oxide; HCC, hepatocellular carcinoma; HGC-27, human gastric cancer cell-27; IGF-1Ra, insulin-like growth factor-1Ra; KD1, kunitz domain 1; MDR, multidrug resistance; MTX, methotrexate; MWNTs, multi-wall carbon nanotubes; MYH9, myosin heavy chain 9; OSA, oxidized sodium alginate; OSCC, oral squamous cell carcinoma; OVA, ovalbumin; pAT_2_, plasmid angiotensin II type 2 receptor; PAT, photoacoustic therapy; PDT, photodynamic therapy; PEI, polyetherimide; PEG, polyethylene glycol; P-gp, p-glycoprotein; PL, peptide lipid; PTEN, phosphatase and tensin homolog; PTT, photothermal therapy; PTX, paclitaxel; ROS, reactive oxygen species; SWNTs, single-wall carbon nanotubes; TME, tumor microenvironment; TNF-α, tumor necrosis factor-alpha; TPGS, D-α-tocopheryl polyethylene glycol succinate; TPP, triphenylphosphine.

**Table 2:** Representative applications of carbon nanosystems in cancer imaging

Type of Carbon nano systems	Imaging approach	Functionalized molecules	Tumor model	*In vitro*/*in vivo*	Ref.
GO	Fluorescence Imaging	PEG, FA	B16F0 melanoma	*In vitro*/*in vivo*	129
	MRI	Gd, Au	HepG2 liver cancer	*In vitro*	130
	MRI	Gd (III)	MCF-7 breast cancer	*In vitro*/*in vivo*	131
	PAI	Cy5.5	H1975 lung cancer	*In vivo*	132
	Raman imaging	Au	Hela cervical cancer	*In vitro*	133
rGO	Radionuclide Imaging	PEG, ^131^I	4T1 breast cancer	*In vitro*/*in vivo*	134
	PET	PEG, 1,4,7-triazacyclononane-1,4,7-triacetic acid, (NOTA),^64^Cu	MCF-7 breast cancer	*In vitro*/*in vivo*	135
NGO	NIRF imaging	PEG	Raji B-cell Burkitt's Lymhoma	*In vitro, in vivo*	136
Fullerene	NIRF imaging	HA	HCT 116 colon cancer	*In vivo*	137
	PAI	D-A antenna, DSPE-mPEG	Hela cervical cancer	*In vivo*	79
SWNTs	Raman Imaging	FA, PEG, Au	KB oral epithelial carcinoma	*In vitro*	138
	MRI	Aspargine-glycine-arginine (NGR) peptide, gadolinium-diethylenetriamine pentaacetic acid	MCF-7 breast cancer	*In vitro*/*in vivo*	139
	PAI	/	Human breast cancer	*In vitro*	140
	Radionuclide Imaging	PDA, PEG	4T1 breast cancer	*In vivo*	141
	NIRF imaging	Phospholipid-PEG	MDA-MB-468 breast cancer	*In vitro*/*in vivo*	142
MWNTs	MRI	FA, GdN	Hela cervical cancer	*In vitro*/*in vivo*	143
	Ultrasonography	Fe_3_O_4_, AuNPs	MCF-7 breast cancer	*In vitro*	144
	PAI	RGD peptide, silica-coated gold nanorods	MGC803 gastric cancer	*In vitro*/*in vivo*	145
	Radionuclide Imaging	Indium-111	C57/B16 melanoma	*In vivo*	146
CQDs	Fluorescence imaging	AS141, Ce6	Hela cervical cancer	*In vitro*	147
	Fluorescence imaging	silica NPs, L-cysteine	HEp-2 laryngeal cancer	*In vitro*	148

Abbreviations: AuNPs, Au nanoparticles; Ce6, chlorin e6; CQDs, carbon quantum dots; FA, folic acid; Gd, gadolinium; GdN, gadolinium nanoparticle; GO, graphene oxide; HA**,** hyaluronic acid; MRI: magnetic resonance imaging; MWNTs: multi-wall carbon nanotubes; NGO, nano graphene oxide; NGR, asparagine-glycine-arginine; NIRF**,** near-infrared fluorescence; NOTA, 1,4,7-triazacyclononane-1,4,7-triacetic acid; PAI, photoacoustic imaging; PDA, polydopamine; PEG, polyethylene glycol; PET, positron emission tomography; rGO, reduced graphene oxide; SWNTs: single-wall carbon nanotubes.

## Abbreviations

AFP: alpha-fetoprotein
APCs: antigen-presenting Cells
BMs: base membranes
BSA: bovine serum albumin
CA 125: cancer antigen 125
CAM: chorioallantoic membrane
C-dots: carbon dots
CEA: carcinoembryonic antigen
Ce6: chlorin e6
CNPs: carbon nanoparticles
CNTs: carbon nanotubes
CQDs: carbon quantum dots
CSCs: cancer stem cells
CS: chitosan
CT: computed tomography
ECM: extracellular matrix
EDA: ethylenediamine
EMT: epithelial-mesenchymal transition
ESCC: esophageal squamous cell carcinoma
FA: folic acid
FRET: fluorescence resonance energy transfer
GCQDs: green carbon quantum dots
GdN: gadolinium nanoparticles
GDY: graphdiyne
GNR: graphene nanoribbons
GO: graphdiyne oxide
GQDs: graphene quantum dots
GSH: glutathione
HCC: hepatocellular carcinoma
IGFR: insulin-like growth factor receptor
ITM: immunosuppressive tumor microenvironment
KD1: kunitz domain 1
MB: molecular beacon
MDR: multidrug resistance
miRNAs: microRNAs
MMP-2: matrix metalloproteinase-2
MRI: magnetic resonance imaging
mRNA: messenger RNA
MTP: mitochondrial transmembrane potential
MTX: methotrexate
MWNTs: multi-wall carbon nanotubes
MYH9: myosin heavy chain 9
NIR: near-infrared
OSA: oxidized sodium alginate
OSCC: oral squamous cell carcinoma
OVA: ovalbumin
PAI: photoacoustic imaging
pAT_2_: plasmid angiotensin II type 2 receptor
PDA: polydopamine
pDNA: plasmid deoxyribonucleic acid
PDT: photodynamic therapy
PEG: polyethylene glycol
PEI: polyetherimide
PET: positron emission tomography
P-gp: P-glycoprotein
PL: peptide lipid
PSCA: prostate stem cell antigen
PSCs: pancreatic stellate cells
PTEN: phosphatase and tensin homolog
PTX: paclitaxel
R848: resiquimod
rGO: reduced graphene oxide
RI: resistance index
RNAi: RNA interference
ROS: reactive oxygen species
SERS: surface enhanced Raman scattering
SiNPs: silica nanoparticles
SL: sucrose laurate
SPECT: single-photon-emission computed tomography
st14: suppressor of tumorigenicity 14
SWNTs: single-wall carbon nanotubes
TAAs: tumor-associated antigens
TAT: thermoacoustic therapy
2D: two-dimensional
TME: tumor microenvironment
TNF-α: tumor necrosis factor-alpha
TPGS: tocopheryl polyethylene glycol succinate
TPP: triphenylphosphine
TTIS: tumor targeted iron sponge
UCPL: upconversion photoluminescence
US: ultrasonography
UV: ultraviolet

## References

[1] Siegel RL, Miller KD, Fuchs HE, Jemal A (2021). Cancer Statistics, 2021. CA Cancer J Clin.

[2] Klein CA (2020). Cancer progression and the invisible phase of metastatic colonization. Nat Rev Cancer.

[3] Chen K, Cao X, Li M, Su Y, Li H, Xie M (2019). A TRAIL-Delivered Lipoprotein-Bioinspired Nanovector Engineering Stem Cell-Based Platform for Inhibition of Lung Metastasis of Melanoma. Theranostics.

[4] Su YJ, Wang TT, Su YN, Li M, Zhou JP, Zhang W (2020). A neutrophil membrane-functionalized black phosphorus riding inflammatory signal for positive feedback and multimode cancer therapy. Mater Horiz.

[5] Tang L, Li J, Zhao Q, Pan T, Zhong H, Wang W (2021). Advanced and Innovative Nano-Systems for Anticancer Targeted Drug Delivery. Pharmaceutics.

[6] Su Y, Liu Y, Xu X, Zhou J, Xu L, Xu X (2018). On-Demand Versatile Prodrug Nanomicelle for Tumor-Specific Bioimaging and Photothermal-Chemo Synergistic Cancer Therapy. ACS Appl Mater Interfaces.

[7] Zhang F, Li M, Su Y, Zhou J, Wang W (2016). A dual-targeting drug co-delivery system for tumor chemo- and gene combined therapy. Mater Sci Eng C Mater Biol Appl.

[8] Li M, Su Y, Zhang F, Chen K, Xu X, Xu L (2018). A dual-targeting reconstituted high density lipoprotein leveraging the synergy of sorafenib and antimiRNA21 for enhanced hepatocellular carcinoma therapy. Acta Biomater.

[9] Mei Y, Tang L, Xiao Q, Zhang Z, Zhang Z, Zang J (2021). Reconstituted high density lipoprotein (rHDL), a versatile drug delivery nanoplatform for tumor targeted therapy. J Mater Chem B.

[10] Tang L, He S, Yin Y, Liu H, Hu J, Cheng J (2021). Combination of Nanomaterials in Cell-Based Drug Delivery Systems for Cancer Treatment. Pharmaceutics.

[11] Augustine S, Singh J, Srivastava M, Sharma M, Das A, Malhotra BD (2017). Recent advances in carbon based nanosystems for cancer theranostics. Biomater Sci.

[12] Saleem J, Wang L, Chen C (2018). Carbon-Based Nanomaterials for Cancer Therapy via Targeting Tumor Microenvironment. Adv Healthc Mater.

[13] Su Y, Hu Y, Wang Y, Xu X, Yuan Y, Li Y (2017). A precision-guided MWNT mediated reawakening the sunk synergy in RAS for anti-angiogenesis lung cancer therapy. Biomaterials.

[14] Wong BS, Yoong SL, Jagusiak A, Panczyk T, Ho HK, Ang WH (2013). Carbon nanotubes for delivery of small molecule drugs. Adv Drug Deliv Rev.

[15] Henna TK, Raphey VR, Sankar R, Ameena Shirin VK, Gangadharappa HV, Pramod K (2020). Carbon nanostructures: The drug and the delivery system for brain disorders. Int J Pharm.

[16] Bartelmess J, Quinn SJ, Giordani S (2015). Carbon nanomaterials: multi-functional agents for biomedical fluorescence and Raman imaging. Chem Soc Rev.

[17] Buskaran K, Hussein MZ, Moklas MAM, Masarudin MJ, Fakurazi S (2021). Graphene Oxide Loaded with Protocatechuic Acid and Chlorogenic Acid Dual Drug Nanodelivery System for Human Hepatocellular Carcinoma Therapeutic Application. Int J Mol Sci.

[18] Hong G, Diao S, Antaris AL, Dai H (2015). Carbon Nanomaterials for Biological Imaging and Nanomedicinal Therapy. Chem Rev.

[19] Tang L, Mei Y, Shen Y, He S, Xiao Q, Yin Y (2021). Nanoparticle-Mediated Targeted Drug Delivery to Remodel Tumor Microenvironment for Cancer Therapy. Int J Nanomedicine.

[20] Li M, Zhang F, Su Y, Zhou J, Wang W (2018). Nanoparticles designed to regulate tumor microenvironment for cancer therapy. Life Sci.

[21] Harisa GI, Faris TM (2019). Direct Drug Targeting into Intracellular Compartments: Issues, Limitations, and Future Outlook. J Membr Biol.

[22] Wang W, Chen K, Su Y, Zhang J, Li M, Zhou J (2018). Lysosome-Independent Intracellular Drug/Gene Codelivery by Lipoprotein-Derived Nanovector for Synergistic Apoptosis-Inducing Cancer-Targeted Therapy. Biomacromolecules.

[23] Kaymak I, Williams KS, Cantor JR, Jones RG (2021). Immunometabolic Interplay in the Tumor Microenvironment. Cancer Cell.

[24] Guo W, Chen Z, Feng X, Shen G, Huang H, Liang Y (2021). Graphene oxide (GO)-based nanosheets with combined chemo/photothermal/photodynamic therapy to overcome gastric cancer (GC) paclitaxel resistance by reducing mitochondria-derived adenosine-triphosphate (ATP). J Nanobiotechnology.

[25] Ou L, Sun T, Liu M, Zhang Y, Zhou Z, Zhan X (2020). Efficient miRNA Inhibitor Delivery with Graphene Oxide-Polyethylenimine to Inhibit Oral Squamous Cell Carcinoma. Int J Nanomedicine.

[26] Jaleel JA, Ashraf SM, Rathinasamy K, Pramod K (2019). Carbon dot festooned and surface passivated graphene-reinforced chitosan construct for tumor-targeted delivery of TNF-alpha gene. Int J Biol Macromol.

[27] Yin Y, Li X, Ma H, Zhang J, Yu D, Zhao R (2021). In Situ Transforming RNA Nanovaccines from Polyethylenimine Functionalized Graphene Oxide Hydrogel for Durable Cancer Immunotherapy. Nano Lett.

[28] Wu B, Li M, Wang L, Iqbal Z, Zhu K, Yang Y (2021). Size-transformable nanohybrids with pH/redox/enzymatic sensitivity for anticancer therapy. J Mater Chem B.

[29] Lee HR, Kim DW, Jones VO, Choi Y, Ferry VE, Geller MA (2021). Sonosensitizer-Functionalized Graphene Nanoribbons for Adhesion Blocking and Sonodynamic Ablation of Ovarian Cancer Spheroids. Adv Healthc Mater.

[30] Zhou X, You M, Wang F, Wang Z, Gao X, Jing C (2021). Multifunctional Graphdiyne-Cerium Oxide Nanozymes Facilitate MicroRNA Delivery and Attenuate Tumor Hypoxia for Highly Efficient Radiotherapy of Esophageal Cancer. Adv Mater.

[31] Xing E, Du Y, Yin J, Chen M, Zhu M, Wen X (2021). Multi-functional Nanodrug Based on a Three-dimensional Framework for Targeted Photo-chemo Synergetic Cancer Therapy. Adv Healthc Mater.

[32] Min H, Qi Y, Zhang Y, Han X, Cheng K, Liu Y (2020). A Graphdiyne Oxide-Based Iron Sponge with Photothermally Enhanced Tumor-Specific Fenton Chemistry. Adv Mater.

[33] Jiang W, Zhang Z, Wang Q, Dou J, Zhao Y, Ma Y (2019). Tumor Reoxygenation and Blood Perfusion Enhanced Photodynamic Therapy using Ultrathin Graphdiyne Oxide Nanosheets. Nano Lett.

[34] Serda M, Ware MJ, Newton JM, Sachdeva S, Krzykawska-Serda M, Nguyen L (2018). Development of photoactive Sweet-C60 for pancreatic cancer stellate cell therapy. Nanomedicine (Lond).

[35] Wang J, Xie L, Wang T, Wu F, Meng J, Liu J (2017). Visible light-switched cytosol release of siRNA by amphiphilic fullerene derivative to enhance RNAi efficacy in vitro and in vivo. Acta Biomater.

[36] Sosnowska M, Kutwin M, Jaworski S, Strojny B, Wierzbicki M, Szczepaniak J (2019). Mechano-signalling, induced by fullerene C60 nanofilms, arrests the cell cycle in the G2/M phase and decreases proliferation of liver cancer cells. Int J Nanomedicine.

[37] Zhou W, Huo J, Yang Y, Zhang X, Li S, Zhao C (2020). Aminated Fullerene Abrogates Cancer Cell Migration by Directly Targeting Myosin Heavy Chain 9. ACS Appl Mater Interfaces.

[38] Li L, Zhen M, Wang H, Sun Z, Jia W, Zhao Z (2020). Functional Gadofullerene Nanoparticles Trigger Robust Cancer Immunotherapy Based on Rebuilding an Immunosuppressive Tumor Microenvironment. Nano Lett.

[39] Zhao Y, Zhao T, Cao Y, Sun J, Zhou Q, Chen H (2021). Temperature-Sensitive Lipid-Coated Carbon Nanotubes for Synergistic Photothermal Therapy and Gene Therapy. ACS Nano.

[40] Lu GH, Shang WT, Deng H, Han ZY, Hu M, Liang XY (2019). Targeting carbon nanotubes based on IGF-1R for photothermal therapy of orthotopic pancreatic cancer guided by optical imaging. Biomaterials.

[41] Wen L, Ding W, Yang S, Xing D (2016). Microwave pumped high-efficient thermoacoustic tumor therapy with single wall carbon nanotubes. Biomaterials.

[42] Al Faraj A, Shaik AS, Al Sayed B, Halwani R, Al Jammaz I (2016). Specific targeting and noninvasive imaging of breast cancer stem cells using single-walled carbon nanotubes as novel multimodality nanoprobes. Nanomedicine (Lond).

[43] Hassan HA, Smyth L, Wang JT, Costa PM, Ratnasothy K, Diebold SS (2016). Dual stimulation of antigen presenting cells using carbon nanotube-based vaccine delivery system for cancer immunotherapy. Biomaterials.

[44] Li G, Pei M, Liu P (2020). pH/Reduction dual-responsive comet-shaped PEGylated CQD-DOX conjugate prodrug: Synthesis and self-assembly as tumor nanotheranostics. Mater Sci Eng C Mater Biol Appl.

[45] Wang L, Wang X, Bhirde A, Cao J, Zeng Y, Huang X (2014). Carbon-dot-based two-photon visible nanocarriers for safe and highly efficient delivery of siRNA and DNA. Adv Healthc Mater.

[46] Zhang Y, Zhang C, Chen J, Liu L, Hu M, Li J (2017). Trackable Mitochondria-Targeting Nanomicellar Loaded with Doxorubicin for Overcoming Drug Resistance. ACS Appl Mater Interfaces.

[47] Chen D, Li B, Lei T, Na D, Nie M, Yang Y (2021). Selective mediation of ovarian cancer SKOV3 cells death by pristine carbon quantum dots/Cu2O composite through targeting matrix metalloproteinases, angiogenic cytokines and cytoskeleton. J Nanobiotechnology.

[48] Hu P, Shang L, Chen J, Chen X, Chen C, Hong W (2020). A nanometer-sized protease inhibitor for precise cancer diagnosis and treatment. J Mater Chem B.

[49] Gu Z, Zhu S, Yan L, Zhao F, Zhao Y (2019). Graphene-Based Smart Platforms for Combined Cancer Therapy. Adv Mater.

[50] Orecchioni M, Cabizza R, Bianco A, Delogu LG (2015). Graphene as cancer theranostic tool: progress and future challenges. Theranostics.

[51] Liu J, Dong J, Zhang T, Peng Q (2018). Graphene-based nanomaterials and their potentials in advanced drug delivery and cancer therapy. J Control Release.

[52] Yang G, Li L, Lee WB, Ng MC (2018). Structure of graphene and its disorders: a review. Sci Technol Adv Mater.

[53] Lu X, Yang L, Yang Z (2020). Photothermal Sensing of Nano-Devices Made of Graphene Materials. Sensors (Basel).

[54] Pang Y, Mai Z, Wang B, Wang L, Wu L, Wang X (2017). Artesunate-modified nano-graphene oxide for chemo-photothermal cancer therapy. Oncotarget.

[55] Cao W, He L, Cao W, Huang X, Jia K, Dai J (2020). Recent progress of graphene oxide as a potential vaccine carrier and adjuvant. Acta Biomater.

[56] Chen X, Liu L, Jiang C (2016). Charge-reversal nanoparticles: novel targeted drug delivery carriers. Acta Pharm Sin B.

[57] Krystek M, Pakulski D, Patroniak V, Gorski M, Szojda L, Ciesielski A (2019). High-Performance Graphene-Based Cementitious Composites. Adv Sci (Weinh).

[58] Islam MS, Renner F, Azizighannad S, Mitra S (2020). Direct incorporation of nano graphene oxide (nGO) into hydrophobic drug crystals for enhanced aqueous dissolution. Colloids Surf B Biointerfaces.

[59] Yang Y, Karakhanova S, Hartwig W, D'Haese JG, Philippov PP, Werner J (2016). Mitochondria and Mitochondrial ROS in Cancer: Novel Targets for Anticancer Therapy. J Cell Physiol.

[60] Gong Y, Yang H, Tian X (2017). Elucidating the mechanism of miRNA-214 in the regulation of gingival carcinoma. Exp Ther Med.

[61] Shankla M, Aksimentiev A (2014). Conformational transitions and stop-and-go nanopore transport of single-stranded DNA on charged graphene. Nat Commun.

[62] Miao L, Li L, Huang Y, Delcassian D, Chahal J, Han J (2019). Delivery of mRNA vaccines with heterocyclic lipids increases anti-tumor efficacy by STING-mediated immune cell activation. Nat Biotechnol.

[63] Minchinton AI, Tannock IF (2006). Drug penetration in solid tumours. Nat Rev Cancer.

[64] Li G, Li Y, Liu H, Guo Y, Li Y, Zhu D (2010). Architecture of graphdiyne nanoscale films. Chem Commun (Camb).

[65] Jin J, Guo M, Liu J, Liu J, Zhou H, Li J (2018). Graphdiyne Nanosheet-Based Drug Delivery Platform for Photothermal/Chemotherapy Combination Treatment of Cancer. ACS Appl Mater Interfaces.

[66] Xie C, Wang N, Li X, Xu G, Huang C (2020). Research on the Preparation of Graphdiyne and Its Derivatives. Chemistry.

[67] Wei W, Zhang X, Zhang S, Wei G, Su Z (2019). Biomedical and bioactive engineered nanomaterials for targeted tumor photothermal therapy: A review. Mater Sci Eng C Mater Biol Appl.

[68] Yu H, Xue Y, Li Y (2019). Graphdiyne and its Assembly Architectures: Synthesis, Functionalization, and Applications. Adv Mater.

[69] Fusco L, Gazzi A, Peng G, Shin Y, Vranic S, Bedognetti D (2020). Graphene and other 2D materials: a multidisciplinary analysis to uncover the hidden potential as cancer theranostics. Theranostics.

[70] Parvin N, Jin Q, Wei Y, Yu R, Zheng B, Huang L (2017). Few-Layer Graphdiyne Nanosheets Applied for Multiplexed Real-Time DNA Detection. Adv Mater.

[71] Li S, Chen Y, Liu H, Wang Y, Liu L, Lv F (2017). Graphdiyne Materials as Nanotransducer for in Vivo Photoacoustic Imaging and Photothermal Therapy of Tumor. Chemistry of Materials.

[72] Revia RA, Stephen ZR, Zhang M (2019). Theranostic Nanoparticles for RNA-Based Cancer Treatment. Acc Chem Res.

[73] Xie J, Gong L, Zhu S, Yong Y, Gu Z, Zhao Y (2019). Emerging Strategies of Nanomaterial-Mediated Tumor Radiosensitization. Adv Mater.

[74] Guo Y, Zhang Y, Ma J, Li Q, Li Y, Zhou X (2018). Light/magnetic hyperthermia triggered drug released from multi-functional thermo-sensitive magnetoliposomes for precise cancer synergetic theranostics. J Control Release.

[75] Tang Z, Zhao P, Wang H, Liu Y, Bu W (2021). Biomedicine Meets Fenton Chemistry. Chem Rev.

[76] Kroto HW, Heath JR, O'Brien SC, Curl RF, Smalley RE (1985). C60: Buckminsterfullerene. Nature.

[77] Jariwala D, Sangwan VK, Lauhon LJ, Marks TJ, Hersam MC (2013). Carbon nanomaterials for electronics, optoelectronics, photovoltaics, and sensing. Chem Soc Rev.

[78] Ding C, Tong L, Feng J, Fu J (2016). Recent Advances in Stimuli-Responsive Release Function Drug Delivery Systems for Tumor Treatment. Molecules.

[79] Shi H, Gu R, Xu W, Huang H, Xue L, Wang W (2019). Near-Infrared Light-Harvesting Fullerene-Based Nanoparticles for Promoted Synergetic Tumor Phototheranostics. ACS Appl Mater Interfaces.

[80] Ye L, Kollie L, Liu X, Guo W, Ying X, Zhu J (2021). Antitumor Activity and Potential Mechanism of Novel Fullerene Derivative Nanoparticles. Molecules.

[81] Wang T, Wang C (2019). Functional Metallofullerene Materials and Their Applications in Nanomedicine, Magnetics, and Electronics. Small.

[82] Zhang H, Hou L, Jiao X, Ji Y, Zhu X, Zhang Z (2015). Transferrin-mediated fullerenes nanoparticles as Fe(2+)-dependent drug vehicles for synergistic anti-tumor efficacy. Biomaterials.

[83] Wang T, Upponi JR, Torchilin VP (2012). Design of multifunctional non-viral gene vectors to overcome physiological barriers: dilemmas and strategies. Int J Pharm.

[84] Soung YH, Nguyen T, Cao H, Lee J, Chung J (2016). Emerging roles of exosomes in cancer invasion and metastasis. BMB Rep.

[85] Wang B, Qi X, Liu J, Zhou R, Lin C, Shangguan J (2019). MYH9 Promotes Growth and Metastasis via Activation of MAPK/AKT Signaling in Colorectal Cancer. J Cancer.

[86] Wu S, Zheng Q, Xing X, Dong Y, Wang Y, You Y (2018). Matrix stiffness-upregulated LOXL2 promotes fibronectin production, MMP9 and CXCL12 expression and BMDCs recruitment to assist pre-metastatic niche formation. J Exp Clin Cancer Res.

[87] Deline AR, Frank BP, Smith CL, Sigmon LR, Wallace AN, Gallagher MJ (2020). Influence of Oxygen-Containing Functional Groups on the Environmental Properties, Transformations, and Toxicity of Carbon Nanotubes. Chem Rev.

[88] Zare H, Ahmadi S, Ghasemi A, Ghanbari M, Rabiee N, Bagherzadeh M (2021). Carbon Nanotubes: Smart Drug/Gene Delivery Carriers. Int J Nanomedicine.

[89] Chen D, Dougherty CA, Zhu K, Hong H (2015). Theranostic applications of carbon nanomaterials in cancer: Focus on imaging and cargo delivery. J Control Release.

[90] Panwar N, Soehartono AM, Chan KK, Zeng S, Xu G, Qu J (2019). Nanocarbons for Biology and Medicine: Sensing, Imaging, and Drug Delivery. Chem Rev.

[91] Li H, Conde J, Guerreiro A, Bernardes GJL (2020). Tetrazine Carbon Nanotubes for Pretargeted In Vivo "Click-to-Release" Bioorthogonal Tumour Imaging. Angew Chem Int Ed Engl.

[92] Tang L, Xiao Q, Mei Y, He S, Zhang Z, Wang R (2021). Insights on functionalized carbon nanotubes for cancer theranostics. J Nanobiotechnology.

[93] Loh KP, Ho D, Chiu GNC, Leong DT, Pastorin G, Chow EK (2018). Clinical Applications of Carbon Nanomaterials in Diagnostics and Therapy. Adv Mater.

[94] Corletto A, Shapter JG (2020). Nanoscale Patterning of Carbon Nanotubes: Techniques, Applications, and Future. Adv Sci (Weinh).

[95] Tang L, Zhang A, Zhang Z, Zhao Q, Li J, Mei Y (2022). Multifunctional inorganic nanomaterials for cancer photoimmunotherapy. Cancer Commun (Lond).

[96] Chen X, Zhang Q, Li J, Yang M, Zhao N, Xu FJ (2018). Rattle-Structured Rough Nanocapsules with in-Situ-Formed Gold Nanorod Cores for Complementary Gene/Chemo/Photothermal Therapy. ACS Nano.

[97] Tang L, Zhang A, Mei Y, Xiao Q, Xu X, Wang W (2021). NIR Light-Triggered Chemo-Phototherapy by ICG Functionalized MWNTs for Synergistic Tumor-Targeted Delivery. Pharmaceutics.

[98] Huang ZQ, Buchsbaum DJ (2009). Monoclonal antibodies in the treatment of pancreatic cancer. Immunotherapy.

[99] Qiao Y, Gou G, Wu F, Jian J, Li X, Hirtz T (2020). Graphene-Based Thermoacoustic Sound Source. ACS Nano.

[100] Mannello F (2013). Understanding breast cancer stem cell heterogeneity: time to move on to a new research paradigm. BMC Med.

[101] Wang L, Zuo X, Xie K, Wei D (2018). The Role of CD44 and Cancer Stem Cells. Methods Mol Biol.

[102] Fu S, Zhao Y, Sun J, Yang T, Zhi D, Zhang E (2021). Integrin alphavbeta3-targeted liposomal drug delivery system for enhanced lung cancer therapy. Colloids Surf B Biointerfaces.

[103] Xu X, Ray R, Gu Y, Ploehn HJ, Gearheart L, Raker K (2004). Electrophoretic analysis and purification of fluorescent single-walled carbon nanotube fragments. J Am Chem Soc.

[104] Li D, Fan Y, Shen M, Banyai I, Shi X (2019). Design of dual drug-loaded dendrimer/carbon dot nanohybrids for fluorescence imaging and enhanced chemotherapy of cancer cells. J Mater Chem B.

[105] Li D, Lin L, Fan Y, Liu L, Shen M, Wu R (2021). Ultrasound-enhanced fluorescence imaging and chemotherapy of multidrug-resistant tumors using multifunctional dendrimer/carbon dot nanohybrids. Bioact Mater.

[106] Guo Y, Shen M, Shi X (2021). Construction of Poly(amidoamine) Dendrimer/Carbon Dot Nanohybrids for Biomedical Applications. Macromol Biosci.

[107] Guo Y, Fan Y, Li G, Wang Z, Shi X, Shen M (2021). "Cluster Bomb" Based on Redox-Responsive Carbon Dot Nanoclusters Coated with Cell Membranes for Enhanced Tumor Theranostics. ACS Appl Mater Interfaces.

[108] Si Q-S, Guo W-Q, Wang H-Z, Liu B-H, Ren N-Q (2020). Carbon quantum dots-based semiconductor preparation methods, applications and mechanisms in environmental contamination. Chin Chem Lett.

[109] Fernando KA, Sahu S, Liu Y, Lewis WK, Guliants EA, Jafariyan A (2015). Carbon quantum dots and applications in photocatalytic energy conversion. ACS Appl Mater Interfaces.

[110] Zhao DL, Chung TS (2018). Applications of carbon quantum dots (CQDs) in membrane technologies: A review. Water Res.

[111] Vasimalai N, Vilas-Boas V, Gallo J, Cerqueira MF, Menendez-Miranda M, Costa-Fernandez JM (2018). Green synthesis of fluorescent carbon dots from spices for in vitro imaging and tumour cell growth inhibition. Beilstein J Nanotechnol.

[112] Tungare K, Bhori M, Racherla KS, Sawant S (2020). Synthesis, characterization and biocompatibility studies of carbon quantum dots from Phoenix dactylifera. 3 Biotech.

[113] Devi P, Saini S, Kim KH (2019). The advanced role of carbon quantum dots in nanomedical applications. Biosens Bioelectron.

[114] Xue B, Yang Y, Sun Y, Fan J, Li X, Zhang Z (2019). Photoluminescent lignin hybridized carbon quantum dots composites for bioimaging applications. Int J Biol Macromol.

[115] Wisniewski M, Czarnecka J, Bolibok P, Swidzinski M, Roszek K (2021). New Insight into the Fluorescence Quenching of Nitrogen-Containing Carbonaceous Quantum Dots-From Surface Chemistry to Biomedical Applications. Materials (Basel).

[116] Dugam S, Nangare S, Patil P, Jadhav N (2021). Carbon dots: A novel trend in pharmaceutical applications. Ann Pharm Fr.

[117] Varma MV, Panchagnula R (2005). Enhanced oral paclitaxel absorption with vitamin E-TPGS: effect on solubility and permeability in vitro, in situ and in vivo. Eur J Pharm Sci.

[118] Li B, Chen D, Wang J, Yan Z, Jiang L, Deliang D (2014). MOFzyme: Intrinsic protease-like activity of Cu-MOF. Sci Rep.

[119] Suzuki M, Kobayashi H, Kanayama N, Saga Y, Suzuki M, Lin CY (2004). Inhibition of tumor invasion by genomic down-regulation of matriptase through suppression of activation of receptor-bound pro-urokinase. J Biol Chem.

[120] Zhao B, Yuan C, Li R, Qu D, Huang M, Ngo JC (2013). Crystal structures of matriptase in complex with its inhibitor hepatocyte growth factor activator inhibitor-1. J Biol Chem.

[121] Schiffman JD, Fisher PG, Gibbs P (2015). Early detection of cancer: past, present, and future. Am Soc Clin Oncol Educ Book.

[122] Witte RS, Tamimi EA (2021). Emerging photoacoustic and thermoacoustic imaging technologies for detecting primary and metastatic cancer and guiding therapy. Clin Exp Metastasis.

[123] Goldman LW (2007). Principles of CT and CT technology. J Nucl Med Technol.

[124] Cartee RE, Hudson JA, Finn-Bodner S (1993). Ultrasonography. Vet Clin North Am Small Anim Pract.

[125] Yousaf T, Dervenoulas G, Politis M (2018). Advances in MRI Methodology. Int Rev Neurobiol.

[126] Umutlu L, Antoch G, Herrmann K, Grueneisen J (2019). PET/MR Imaging of the Female Pelvis. Semin Nucl Med.

[127] Weber W (2020). Clinical PET/MR. Recent Results Cancer Res.

[128] Attia ABE, Balasundaram G, Moothanchery M, Dinish US, Bi R, Ntziachristos V (2019). A review of clinical photoacoustic imaging: Current and future trends. Photoacoustics.

[129] Kalluru P, Vankayala R, Chiang CS, Hwang KC (2016). Nano-graphene oxide-mediated In vivo fluorescence imaging and bimodal photodynamic and photothermal destruction of tumors. Biomaterials.

[130] Usman MS, Hussein MZ, Fakurazi S, Masarudin MJ, Ahmad Saad FF (2018). A bimodal theranostic nanodelivery system based on [graphene oxide-chlorogenic acid-gadolinium/gold] nanoparticles. PLoS One.

[131] Shi J, Wang B, Chen Z, Liu W, Pan J, Hou L (2016). A Multi-Functional Tumor Theranostic Nanoplatform for MRI Guided Photothermal-Chemotherapy. Pharm Res.

[132] Nie L, Huang P, Li W, Yan X, Jin A, Wang Z (2014). Early-stage imaging of nanocarrier-enhanced chemotherapy response in living subjects by scalable photoacoustic microscopy. ACS Nano.

[133] Ma X, Qu Q, Zhao Y, Luo Z, Zhao Y, Ng KW (2013). Graphene oxide wrapped gold nanoparticles for intracellular Raman imaging and drug delivery. J Mater Chem B.

[134] Chen L, Zhong X, Yi X, Huang M, Ning P, Liu T (2015). Radionuclide (131)I labeled reduced graphene oxide for nuclear imaging guided combined radio- and photothermal therapy of cancer. Biomaterials.

[135] Shi S, Yang K, Hong H, Valdovinos HF, Nayak TR, Zhang Y (2013). Tumor vasculature targeting and imaging in living mice with reduced graphene oxide. Biomaterials.

[136] Sun X, Liu Z, Welsher K, Robinson JT, Goodwin A, Zaric S (2008). Nano-Graphene Oxide for Cellular Imaging and Drug Delivery. Nano Res.

[137] Kwag DS, Park K, Oh KT, Lee ES (2013). Hyaluronated fullerenes with photoluminescent and antitumoral activity. Chem Commun (Camb).

[138] Wang X, Wang C, Cheng L, Lee ST, Liu Z (2012). Noble metal coated single-walled carbon nanotubes for applications in surface enhanced Raman scattering imaging and photothermal therapy. J Am Chem Soc.

[139] Yan C, Chen C, Hou L, Zhang H, Che Y, Qi Y (2017). Single-walled carbon nanotube-loaded doxorubicin and Gd-DTPA for targeted drug delivery and magnetic resonance imaging. J Drug Target.

[140] Avti PK, Hu S, Favazza C, Mikos AG, Jansen JA, Shroyer KR (2012). Detection, mapping, and quantification of single walled carbon nanotubes in histological specimens with photoacoustic microscopy. PLoS One.

[141] Zhao H, Chao Y, Liu J, Huang J, Pan J, Guo W (2016). Polydopamine Coated Single-Walled Carbon Nanotubes as a Versatile Platform with Radionuclide Labeling for Multimodal Tumor Imaging and Therapy. Theranostics.

[142] Welsher K, Liu Z, Sherlock SP, Robinson JT, Chen Z, Daranciang D (2009). A route to brightly fluorescent carbon nanotubes for near-infrared imaging in mice. Nat Nanotechnol.

[143] Zhang M, Wang W, Cui Y, Zhou N, Shen J (2018). Magnetofluorescent Carbon Quantum Dot Decorated Multiwalled Carbon Nanotubes for Dual-Modal Targeted Imaging in Chemo-Photothermal Synergistic Therapy. ACS Biomater Sci Eng.

[144] Saghatchi F, Mohseni-Dargah M, Akbari-Birgani S, Saghatchi S, Kaboudin B (2020). Cancer Therapy and Imaging Through Functionalized Carbon Nanotubes Decorated with Magnetite and Gold Nanoparticles as a Multimodal Tool. Appl Biochem Biotechnol.

[145] Wang C, Bao C, Liang S, Fu H, Wang K, Deng M (2014). RGD-conjugated silica-coated gold nanorods on the surface of carbon nanotubes for targeted photoacoustic imaging of gastric cancer. Nanoscale Res Lett.

[146] Wang JT, Fabbro C, Venturelli E, Menard-Moyon C, Chaloin O, Da Ros T (2014). The relationship between the diameter of chemically-functionalized multi-walled carbon nanotubes and their organ biodistribution profiles in vivo. Biomaterials.

[147] Shen Y, Wu T, Wang Y, Zhang SL, Zhao X, Chen HY (2021). Nucleolin-Targeted Ratiometric Fluorescent Carbon Dots with a Remarkably Large Emission Wavelength Shift for Precise Imaging of Cathepsin B in Living Cancer Cells. Anal Chem.

[148] Li RS, Gao PF, Zhang HZ, Zheng LL, Li CM, Wang J (2017). Chiral nanoprobes for targeting and long-term imaging of the Golgi apparatus. Chem Sci.

[149] P KS, Bathula C, K NC, Das M (2020). Usage of Graphene Oxide in Fluorescence Quenching-Linked Immunosorbent Assay for the Detection of Cry2Ab Protein Present in Transgenic Plants. J Agric Food Chem.

[150] Derfus AM, Chan WCW, Bhatia SN (2004). Probing the Cytotoxicity Of Semiconductor Quantum Dots. Nano Lett.

[151] Yang L, Liu B, Wang M, Li J, Pan W, Gao X (2018). A Highly Sensitive Strategy for Fluorescence Imaging of MicroRNA in Living Cells and in Vivo Based on Graphene Oxide-Enhanced Signal Molecules Quenching of Molecular Beacon. ACS Appl Mater Interfaces.

[152] Hu D, Zhang J, Gao G, Sheng Z, Cui H, Cai L (2016). Indocyanine Green-Loaded Polydopamine-Reduced Graphene Oxide Nanocomposites with Amplifying Photoacoustic and Photothermal Effects for Cancer Theranostics. Theranostics.

[153] Liu H, Li C, Qian Y, Hu L, Fang J, Tong W (2020). Magnetic-induced graphene quantum dots for imaging-guided photothermal therapy in the second near-infrared window. Biomaterials.

[154] Su X, Chan C, Shi J, Tsang MK, Pan Y, Cheng C (2017). A graphene quantum dot@Fe3O4@SiO2 based nanoprobe for drug delivery sensing and dual-modal fluorescence and MRI imaging in cancer cells. Biosens Bioelectron.

[155] Sanginario A, Miccoli B, Demarchi D (2017). Carbon Nanotubes as an Effective Opportunity for Cancer Diagnosis and Treatment. Biosensors (Basel).

[156] Lovell TC, Bolton SG, Kenison JP, Shangguan J, Otteson CE, Civitci F (2021). Subcellular Targeted Nanohoop for One- and Two-Photon Live Cell Imaging. ACS Nano.

[157] Chen Y-C, Young RJ, Macpherson JV, Wilson NR (2011). Silver-decorated carbon nanotube networks as SERS substrates. Journal of Raman Spectroscopy.

[158] Zheng XT, Ananthanarayanan A, Luo KQ, Chen P (2015). Glowing graphene quantum dots and carbon dots: properties, syntheses, and biological applications. Small.

[159] Bhalla N, Jolly P, Formisano N, Estrela P (2016). Introduction to biosensors. Essays Biochem.

[160] Chikkaveeraiah BV, Bhirde AA, Morgan NY, Eden HS, Chen X (2012). Electrochemical immunosensors for detection of cancer protein biomarkers. ACS Nano.

[161] Kim TH, Lee D, Choi JW (2017). Live cell biosensing platforms using graphene-based hybrid nanomaterials. Biosens Bioelectron.

[162] Damborsky P, Svitel J, Katrlik J (2016). Optical biosensors. Essays Biochem.

[163] Hogdall E (2008). Cancer antigen 125 and prognosis. Curr Opin Obstet Gynecol.

[164] Samadi Pakchin P, Fathi M, Ghanbari H, Saber R, Omidi Y (2020). A novel electrochemical immunosensor for ultrasensitive detection of CA125 in ovarian cancer. Biosens Bioelectron.

[165] Biswas S, Lan Q, Xie Y, Sun X, Wang Y (2021). Label-Free Electrochemical Immunosensor for Ultrasensitive Detection of Carbohydrate Antigen 125 Based on Antibody-Immobilized Biocompatible MOF-808/CNT. ACS Appl Mater Interfaces.

[166] Chen L, Heikkinen L, Wang C, Yang Y, Sun H, Wong G (2019). Trends in the development of miRNA bioinformatics tools. Brief Bioinform.

[167] Zhou L, Wang T, Bai Y, Li Y, Qiu J, Yu W (2020). Dual-amplified strategy for ultrasensitive electrochemical biosensor based on click chemistry-mediated enzyme-assisted target recycling and functionalized fullerene nanoparticles in the detection of microRNA-141. Biosens Bioelectron.

[168] Zhang Y, Li N, Ma W, Yang M, Hou C, Luo X (2021). Ultrasensitive detection of microRNA-21 by using specific interaction of antimonene with RNA as electrochemical biosensor. Bioelectrochemistry.

[169] Liao C, Li Y, Tjong SC (2018). Graphene Nanomaterials: Synthesis, Biocompatibility, and Cytotoxicity. Int J Mol Sci.

[170] Devasena T, Francis AP, Ramaprabhu S (2021). Toxicity of Graphene: An Update. Rev Environ Contam Toxicol.

[171] Zhao C, Song X, Liu Y, Fu Y, Ye L, Wang N (2020). Synthesis of graphene quantum dots and their applications in drug delivery. J Nanobiotechnology.

[172] Malhotra N, Audira G, Castillo AL, Siregar P, Ruallo JMS, Roldan MJ (2021). An Update Report on the Biosafety and Potential Toxicity of Fullerene-Based Nanomaterials toward Aquatic Animals. Oxid Med Cell Longev.

[173] Dong J, Ma Q (2015). Advances in mechanisms and signaling pathways of carbon nanotube toxicity. Nanotoxicology.

[174] Chen M, Qin X, Zeng G (2017). Biodegradation of Carbon Nanotubes, Graphene, and Their Derivatives. Trends Biotechnol.

[175] Liu J, Chen C, Zhao Y (2019). Progress and Prospects of Graphdiyne-Based Materials in Biomedical Applications. Adv Mater.

[176] Lalwani G, D'Agati M, Khan AM, Sitharaman B (2016). Toxicology of graphene-based nanomaterials. Adv Drug Deliv Rev.

[177] Cao Y, Xiao W, Li S, Qiu D (2021). A comparative study of toxicity of graphdiyne and graphene oxide to human umbilical vein endothelial cells. J Appl Toxicol.

[178] Wang L, Li W, Yin L, Liu Y, Guo H, Lai J (2020). Full-color fluorescent carbon quantum dots. Sci Adv.

[179] Mohammadinejad R, Dadashzadeh A, Moghassemi S, Ashrafizadeh M, Dehshahri A, Pardakhty A (2019). Shedding light on gene therapy: Carbon dots for the minimally invasive image-guided delivery of plasmids and noncoding RNAs - A review. J Adv Res.

[180] Liu Y, Gou H, Huang X, Zhang G, Xi K, Jia X (2020). Rational synthesis of highly efficient ultra-narrow red-emitting carbon quantum dots for NIR-II two-photon bioimaging. Nanoscale.

[181] Qi H, Teng M, Liu M, Liu S, Li J, Yu H (2019). Biomass-derived nitrogen-doped carbon quantum dots: highly selective fluorescent probe for detecting Fe(3+) ions and tetracyclines. J Colloid Interface Sci.

[182] Phillips E, Penate-Medina O, Zanzonico PB, Carvajal RD, Mohan P, Ye Y (2014). Clinical translation of an ultrasmall inorganic optical-PET imaging nanoparticle probe. Sci Transl Med.

[183] Juthani R, Madajewski B, Yoo B, Zhang L, Chen PM, Chen F (2020). Ultrasmall Core-Shell Silica Nanoparticles for Precision Drug Delivery in a High-Grade Malignant Brain Tumor Model. Clin Cancer Res.

[184] Chen F, Ma K, Zhang L, Madajewski B, Turker MZ, Gallazzi F (2019). Ultrasmall Renally Clearable Silica Nanoparticles Target Prostate Cancer. ACS Appl Mater Interfaces.

[185] Madajewski B, Chen F, Yoo B, Turker MZ, Ma K, Zhang L (2020). Molecular Engineering of Ultrasmall Silica Nanoparticle-Drug Conjugates as Lung Cancer Therapeutics. Clin Cancer Res.

[186] Zhang X, Chen F, Turker MZ, Ma K, Zanzonico P, Gallazzi F (2020). Targeted melanoma radiotherapy using ultrasmall (177)Lu-labeled alpha-melanocyte stimulating hormone-functionalized core-shell silica nanoparticles. Biomaterials.

[187] Li J, Deng X, Wang L, Liu J, Xu K (2020). Clinical application of carbon nanoparticles in lymphatic mapping during colorectal cancer surgeries: A systematic review and meta-analysis. Dig Liver Dis.

[188] Li Z, Ao S, Bu Z, Wu A, Wu X, Shan F (2016). Clinical study of harvesting lymph nodes with carbon nanoparticles in advanced gastric cancer: a prospective randomized trial. World J Surg Oncol.

[189] Xu XF, Gu J (2017). The application of carbon nanoparticles in the lymph node biopsy of cN0 papillary thyroid carcinoma: A randomized controlled clinical trial. Asian J Surg.

[190] Du X, Zhao C, Zhou M, Ma T, Huang H, Jaroniec M (2017). Hollow Carbon Nanospheres with Tunable Hierarchical Pores for Drug, Gene, and Photothermal Synergistic Treatment. Small.

[191] Wang L, Yan L, Liu J, Chen C, Zhao Y (2018). Quantification of Nanomaterial/Nanomedicine Trafficking in Vivo. Anal Chem.

